# Comparative efficacy of 5 non-pharmacological therapies for adults with post-stroke cognitive impairment: A Bayesian network analysis based on 55 randomized controlled trials

**DOI:** 10.3389/fneur.2022.977518

**Published:** 2022-09-28

**Authors:** Zhendong Li, Lei Yang, Hangjian Qiu, Xiaoqian Wang, Chengcheng Zhang, Yuejuan Zhang

**Affiliations:** ^1^Department of Nursing, The First Affiliated Hospital of Hunan University of Chinese Medicine, Changsha, China; ^2^School of Nursing, Hunan University of Chinese Medicine, Changsha, China

**Keywords:** cognitive function, stroke, post-stroke cognitive impairment (PSCI), non-pharmacological therapies, network meta-analysis (NMA)

## Abstract

**Background:**

As a common sequela after stroke, cognitive impairment negatively impacts patients' activities of daily living and overall rehabilitation. Non-pharmacological therapies have recently drawn widespread attention for their potential in improving cognitive function. However, the optimal choice of non-pharmacological therapies for post-stroke cognitive impairment (PSCI) is still unclear. Hence, in this study, we compared and ranked 5 non-pharmacological therapies for PSCI with a Bayesian Network Meta-analysis (NMA), to offer a foundation for clinical treatment decision-making.

**Methods:**

PubMed, EMBASE, Web of Science, Cochrane Central Register of Controlled Trials, Chinese Biomedical Medicine, China National Knowledge Infrastructure, Wangfang Database, and China Science and Technology Journal Database were searched from database inception to December 31, 2021, to collect Randomized Controlled Trials for PSCI. All of the studies were assessed (according to Cochrane Handbook for Systematic Reviews) and then data were extracted by two researchers separately. Pairwise meta-analysis for direct comparisons was performed using Revman. NMA of Bayesian hierarchical model was performed by WinBUGS and ADDIS. STATA was used to construct network evidence plots and funnel plots.

**Results:**

A total of 55 trials (53 Two-arm trials and 2 Three-arm trials) with 3,092 individuals were included in this study. In the pair-wise meta-analysis, Transcranial Magnetic Stimulation (TMS), Virtual Reality Exposure Therapy (VR), Computer-assisted cognitive rehabilitation (CA), Transcranial Direct Current Stimulation (tDCS), and Acupuncture were superior to normal cognition training in terms of MoCA, MMSE, and BI outcomes. Bayesian NMA showed that the MoCA outcome ranked Acupuncture (84.7%) as the best therapy and TMS (79.7%) as the second. The MMSE outcome ranked TMS (76.1%) as the best therapy and Acupuncture as the second (72.1%). For BI outcome, TMS (89.1%) ranked the best.

**Conclusions:**

TMS and Acupuncture had a better effect on improving cognitive function in post-stroke patients according to our Bayesian NMA. However, this conclusion still needs to be confirmed with large sample size and high-quality randomized controlled trials.

**Registration:**

https://inplasy.com (No. INPLASY202260036).

## Introduction

Post-stroke cognitive impairment (PSCI) is a common comorbidity of stroke, and the prevalence of it varies enormously across studies (17.6–83%), depending on the time of assessment, the study environment, the demographic variables, and the numerous cognitive tests and cut-offs that were utilized (1). PSCI is defined as a clinical syndrome characterized by any sort of cognitive neurodegeneration after stroke, ranging from mild impairment to a more severe form: post-stroke dementia (2, 3). Disruptions in advanced brain functions such as attention, language, memory, executive, and visuospatial function are the most common symptoms of PSCI, which not only have a negative impact on patients' activities of daily living and overall rehabilitation (4–6) but also linked closely to a higher risk of recurrent ischemic stroke (7) and a lower 5-year survival rate (2). In addition, the ongoing care and support needs required by PSCI patients are closely related to the increased physical and psychological burden of family caregivers (8) and the medical and economic burden on society (9). To sum up, PSCI has become a major public health concern that has to be addressed promptly as the great burden of stroke continues to climb (10, 11).

Currently, pharmaceutical interventions such as Acetylcholinesterase inhibitors, memantine, galantamine, etc., which are mainly approved for use in Alzheimer's disease have shown some clinical benefits in vascular dementia (12, 13). Unfortunately, a recent study revealed that little evidence demonstrates they helped symptoms or slowed dementia progression down in PSCI patients (14). On the contrary, side effects and adverse reactions such as gastrointestinal issues (diarrhea or constipation), headaches, dizziness, and so on, do exist in pharmaceutical interventions (15). Therefore, non-pharmacological therapies such as Transcranial Magnetic Stimulation (TMS) (16), Transcranial Direct Current Stimulation (tDCS) (17), Computer-assisted cognitive rehabilitation (CA) (18), Virtual Reality Exposure Therapy (VR) (19), and Acupuncture (20), which have been found have a positive impact on cognitive function of PSCI patients in several systematic review and meta-analysis, have gradually aroused people's attention (21).

However, due to a lack of manpower and resources, most studies to date have only compared individual therapy to traditional cognition training or, at most, two therapies. Direct comparisons provide little useful information for determining which therapy is more appropriate for PSCI patients. It is obvious that a deeper exploration to assess the relative value between different interventions will be greatly helpful for medical decisions and the rehabilitation of PSCI patients. Network meta-analysis is an extension of pairwise meta-analysis that allows data from multiple clinical trials evaluating at least two treatments to be pooled. The incorporation of both direct and indirect information strengthens inferences about each treatment's relative efficacy (22, 23).

Therefore, in the present study, we included 55 RCTs and used Bayesian Network Meta-analysis (NMA) to assess and rank the efficacy of the 5 different alternative strategies listed above, in order to find the best treatment plan for PSCI patients and to provide an evidence-based foundation for clinical treatments decision-making.

## Materials and methods

This study followed the Preferred Reporting Items for Systematic Reviews and Meta-Analyses (PRISMA) extension statement for Network Meta-Analyses (Supplementary Material), and the study protocol has been registered on the INPLASY (Registration number: INPLASY202260036).

### Search strategy

Four English databases (EMBASE, Web of Science, PubMed, Cochrane Central Register of Controlled Trials) and four Chinese databases [China National Knowledge Infrastructure (CNKI), Wangfang Database, China Science and Technology Journal Database, and Chinese Biomedical Medicine (CBM)] were comprehensive searched systematically. MeSH terms, subject words, and keywords such as “Stroke,” “Cerebrovascular Accident,” “Brain Ischemia,” “Cognition Disorders,” “Cognitive Impairment,” “Cognitive Dysfunction,” “Transcranial Magnetic Stimulation,” “Transcranial Direct Current Stimulation,” “Transcranial Direct Current Stimulation,” “Virtual Reality,” “Computer-assisted rehabilitation,” and “Randomized controlled trial” were retrieved to identify potentially eligible studies. The retrieval time was specified from the database's inception to December 31, 2021, and the languages were limited to English and Chinese. We also looked through the references in the included literature to see if there were any other research that fit the criteria. Supplementary Table 1 contains a list of the comprehensive search strategies.

### Eligibility and exclusion criteria

The following criteria were used to select literature: (1) Study design: randomized controlled trials (RCTs); (2) Participants: Adults, regardless of nationality, ethnicity, sex, age, or educational background, who have experienced an ischemic or hemorrhagic stroke recently or in the past, and whose diagnosis was made in accordance with well-defined or globally accepted diagnostic criteria. (3) Intervention and control measures: The experimental group underwent non-pharmaceutical treatments such as acupuncture, VR, TMS, tDCS, or CA. The interventions of the control group consisted of normal rehabilitation (NOR), which is a catch-all term for traditional rehabilitation mixed with cognitive training. Other therapies indicated above but distinct from those used in the intervention group are also included. (4) Outcome indicators: Both the Mini-Mental State Examination (MMSE) and the Montreal Cognitive Assessment Scale (MoCA), which have been used extensively to measure cognitive function, were utilized as the principal measures of cognitive performance. Lower MMSE and MoCA scores are indicative of impaired cognitive function. The Barthel Index (BI) was utilized to evaluate functional independence in activity of daily living as a secondary outcome indicator. A lower BI score suggests a reduced capacity for daily life.

Literature that met the following characteristics was excluded: (1) Studies in which the manner of intervention or control is unclear, or in which drugs that may treat cognitive impairment are used in combination. (2) Studies in which the intervention combined two or more of the aforementioned non-pharmacological therapies in a single intervention. (3) Studies with insufficient data on the results that could not be gathered. (4) Repeated studies, clinical protocols, case reports, animal studies, reviewed articles, and non-randomized controlled trials. (5) The language of studies is not English or Chinese.

### Data extraction

Data were retrieved from the publications by two researchers who reviewed them separately. A standard form table constructed by Microsoft Excel 2019 which includes publication information (authors, publish date), demographic data (gender, age, sample size, the duration of disease), intervention measures, the course of treatment, and outcomes (MOCA, MMSE, BI) was used to manage the data. Due to the possibility of variation in baseline conditions for MoCA, MMSE, and BI among studies, the outcome data finally included in the analysis was approximated using the following formula, as suggested by the Cochrane Handbook for Systematic Reviews of Interventions (version 5.1). And r, the correlation coefficient, has a value of 0.5 in this case.


(1) $$\begin{array}{l} {\bar{\chi }}_{\text{change}}={\bar{\chi }}_{\text{post-treatment}}-{\bar{\chi }}_{\text{change}} \end{array}$$

(2) $$\begin{array}{l} SD_{\text{change}}=\sqrt{{\left(SD_{\text{baseline}}\right)}^{2}+{\left(SD_{\text{post-treatment}}\right)}^{2}-2\times r\times SD_{\text{baseline}}\times SD_{\text{post-treatment}}} \end{array}$$

### Quality assessment

Studies were evaluated for quality using a technique to identify and quantify the potential for bias, as detailed in the Cochrane Handbook for Systematic Reviews of Interventions. Two researchers independently examined each other's work after data extraction and quality evaluation, while a third researcher dealt with any differences of opinion.

### Statistical analysis

Revman (version 5.4, Cochrane Collaboration, Oxford, UK) was used to conduct pairwise meta-analyses for the purpose of making side-by-side comparisons. I-square (I^2^) and *P*-values for the test of heterogeneity were used to determine the degree of heterogeneity between the results. To be more precise, we used fixed-effects models when I^2^ < 50% and *p* > 0.1, and we used random-effects models otherwise. As ways to measure the effects, the mean differences (MD) and 95% confidence interval (CI) were calculated.

WinBUGS (version 1.4.3) and the Aggregate Data Drug Information System (ADDIS, version 1.16.5) were used for the Bayesian framework network meta-analysis. Markov chain Monte Carlo (MCMC) was used to calculate the model with the following parameters: four chains, 50,000 sample iterations, 20,000 burns, and a lean interval of 10. For the purpose of evaluating the model's convergence, the potential size reduction factor (PSRF) was employed. Convergence of a model is better when the PSRF is closer to 1. Considering the anticipated heterogeneity, a random-effects model was used to synthesize study effect sizes. The combined results were presented as MD and 95% CI. If the 95% CI of MD did not contain 0, then the MD was regarded to suggest a statistically significant difference. To provide a probability ranking to the various interventions of each outcome, the surface under the cumulative ranking area (SUCRA) was calculated. The SUCRA values might be anything from 0 to 100%, with larger values suggesting more effectiveness. Further, publication bias and small study effects for each outcome in the included RCTs were evaluated using comparison-adjusted funnel plots generated in STATA software (version 5.2).

Distributional comparisons of clinical data were used to test the transitivity assumption (age, sample size, publication year, etc.), which could be modifiers of treatment efficacy. Heterogeneity was assessed with common tau^2^ statistics and predictive intervals, and sensitivity analysis was used to detect potential studies that increase heterogeneity significantly. We used a node-splitting model for the analysis of the inconsistency test, and the results suggest no statistically significant difference between direct and indirect comparisons when *p* < 0.05. What is more, a loop-specific inconsistency test was performed, in which the 95% CI included zero, indicating good consistency between direct and indirect evidence. Furthermore, determining whether or not two models (consistent and inconsistent) are well-fit was done using the deviance information criterion (DIC).

## Results

### Literature selection

**Figure 1:**
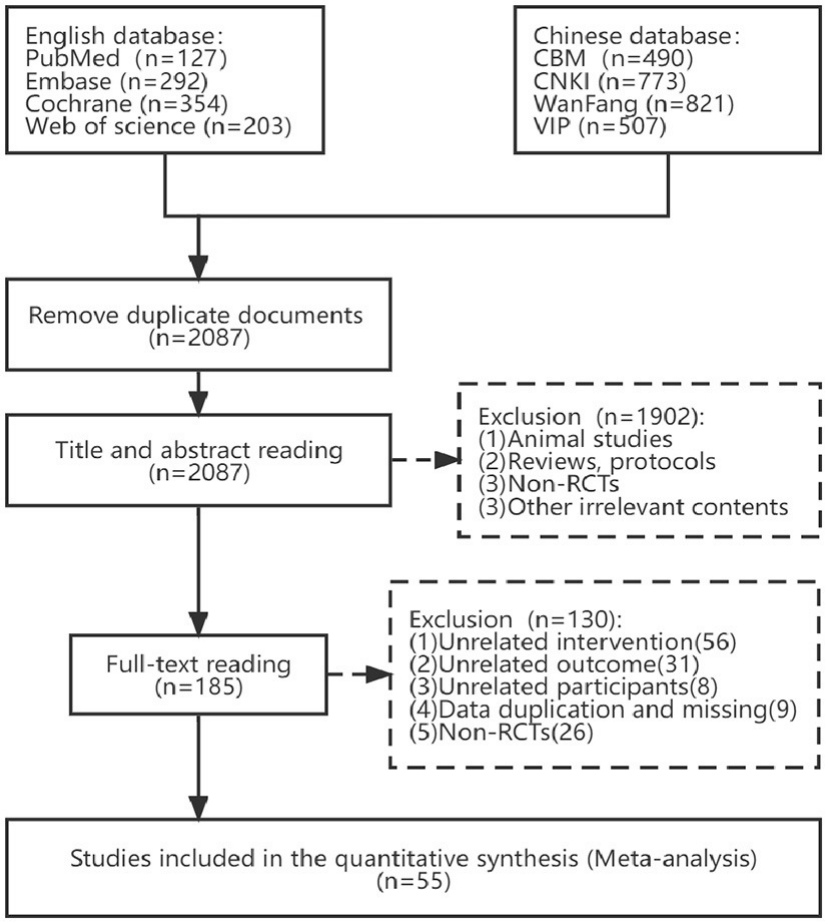
Flow diagram of eligible studies selection process. CBM, Chinese Biomedical Literature Service System; CNKI, China National Knowledge Infrastructure; WanFang, WanFang Knowledge Service platform; VIP, Chinese Scientific Journals Database; *n*, number of publications.

From those 8 databases, we were able to compile a total of 3,567 articles that met our criteria. Once duplicates were taken out, there were still 2,087 articles. Two independent reviewers then screened the titles and abstracts, excluding 1,902 papers that did not meet the inclusion criteria (non-randomized controlled trials, animal studies, case reports, reviews, procedures, and studies that were manifestly irrelevant). By reviewing the remaining articles' entire texts, we were able to weed out another 130 that did not meet our inclusion criterion, including 26 Non-RCTs, 56 unrelated interventions, 31 unrelated outcomes, 8 Non-post-stroke participants, 6 data duplication, and 3 data missing. Finally, 55 published RCTs were included in this NMA. Figure 1 shows a thorough flowchart of the article-screening procedure.

### Study characteristics

Fifty-five articles met the criteria for inclusion; 53 were randomized controlled trials (RCTs) with two arms and 2 were RCTs with three arms. There were a total of 3,092 patients included in the sample (1,496 in the control group and 1,596 in the treatment group). These studies were from China (45), Portugal (4), Korea (3), Russia (1), Australia (2), and Italy (1) and were published from 2008 to 2021. There were 3 studies that only provided the overall gender ratio, 3 studies that did not give patient age, and 2 studies that did not report treatment courses. There was a wide range in length of therapy, from 2 weeks to 12 weeks. There were 33 studies that reported MOCA results, 35 that provided MMSE results, and 23 that reported BI results. Supplementary Table 2 provides a comprehensive summary of relevant research.

### Quality evaluation

**Figure 2:**
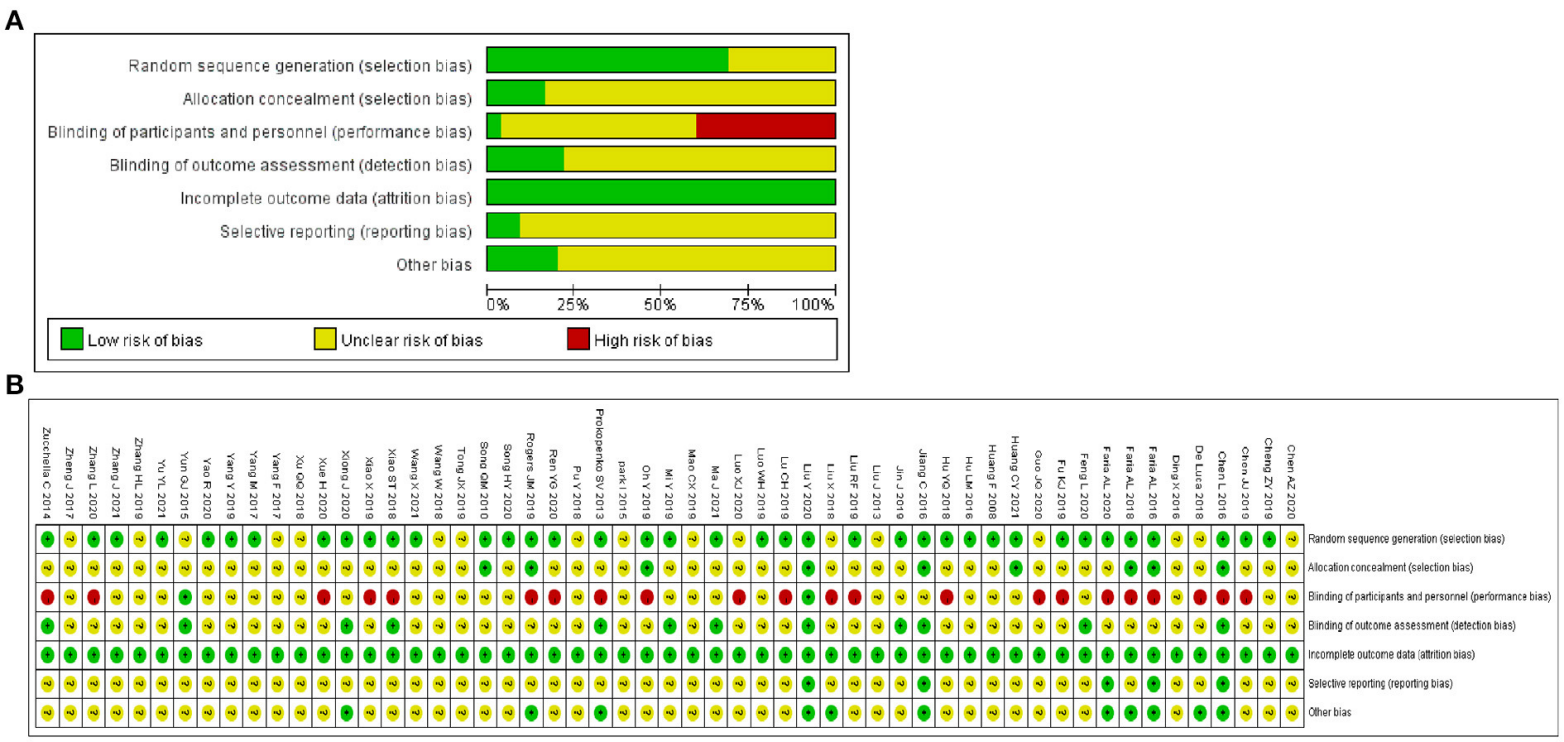
Quality assessment of selected studies by the Cochrane risk of bias tool. **(A)** Risk of bias graph: review authors' judgments about each risk of bias item presented as percentages across all included studies. **(B)** Risk of bias summary: review authors' judgments about each risk of bias item for each included study.

For Random sequence generation, 24 studies reporting the use of a random number table and 14 studies reporting the use of network programming tools were assigned a low risk of bias, and 17 studies not reporting how randomization was performed were assigned an unclear risk of bias. For Allocation concealment, there were 9 studies that met the criteria and were assigned a low risk of bias. For the Blinding of participants and personnel, 2 trials mentioned single blindness and were assigned a low risk of bias, other 21 studies in which intervention measures involving VR and CA were assigned a high risk of bias due to the inability to be blinded. For the Blinding of outcome assessment, 12 trials were assigned a low risk of bias. For Incomplete outcome data, all studies were assigned a low risk of bias as no studies reported severe cases dropped. For Selective reporting, 5 trials that mentioned the study protocol were assigned a low risk of bias. For Other bias, 11 trials that reported disclosure of conflict of interest were assigned a low risk of bias. Figure 2 depicts the summary risk of bias for selected studies.

### Pairwise meta-analysis

Following the synthesis of studies that had the same treatments and outcomes, we carried out eight direct pairwise meta-analyses to compare the MOCA score, 9 to compare the MMSE score, and 6 to compare the BI score, respectively, which can be summarily seen in Table 1. As for the MOCA outcome, TMS (MD = 3.42, 95% CI: 1.86–4.98), tDCS (MD = 2.89, 95% CI: 1.15–4.63), VR (MD = 0.95, 95% CI: 0.09–1.81), CA (MD = 2.17, 95% CI: 0.74–3.60) and Acupuncture (MD = 3.70, 95% CI: 1.51–5.89) were more efficient than NOR. However, there was no statistical difference in efficacy between Acupuncture and CA, tDCS and CA. For MMSE score, TMS (MD = 2.27, 95% CI: 0.18–4.36), tDCS (MD = 1.37, 95% CI: 0.13–2.61), VR (MD = 1.68, 95% CI: 0.49, 2.87) and Acupuncture (MD = 2.31, 95% CI: 0.65–3.97) were more efficient than NOR. However, there was no statistical difference in efficacy between CA and NOR, ACU and CA, tDCS and CA, TMS and CA, VR and ACU. For BI score, TMS (MD = 11.22, 95% CI: 2.53–19.90), tDCS (MD = 10.46, 95% CI: 8.29–12.64), VR (MD = 5.52, 95% CI: 4.24–6.80), CA (MD = 5.44, 95% CI: 2.78, 8.11) and Acupuncture (MD = 9.86, 95% CI: 6.22–13.50) were more efficient than NOR. However, there was no statistical difference in efficacy between VR and Acupuncture. The detailed forest plots of the pairwise meta-analysis results are shown in Supplementary Figures 1–3.

**Table 1:** Pairwise meta-analysis.

**Comparison**	**MD (95% CI)**	**Number of patients**	**Number of studies**	**Heterogeneity test**	
				**I^2^ (%)**	***P*-value**
**MOCA**					
TMS - NOR	**3.42 (1.86, 4.98)**	572	8	92	<0.0001
tDCS - NOR	**2.89 (1.15, 4.63)**	212	4	93	<0.0001
VR - NOR	**0.95 (0.09, 1.81)**	221	6	23	0.26
CA - NOR	**2.17 (0.74, 3.60)**	569	8	85	<0.0001
ACU - NOR	**3.70 (1.51, 5.89)**	709	7	94	<0.0001
ACU - CA	0.06 (−2.28, 2.4)	103	1	–	–
tDCS - CA	0.83 (−1.17, 2.83)	64	1	–	–
**MMSE**					
TMS - NOR	**2.27 (0.18, 4.36)**	341	5	93	<0.0001
tDCS - NOR	**1.37 (0.13, 2.61)**	107	2	0	0.44
VR - NOR	**1.68 (0.49, 2.87)**	403	8	75	0.0003
CA - NOR	0.73 (−1.81, 3.26)	339	5	89	<0.0001
ACU - NOR	**2.31 (0.65, 3.97)**	1,382	15	97	<0.0001
ACU - CA	0.22 (−1.50, 1.94)	103	1	–	–
tDCS - CA	−0.17 (−2.07, 1.73)	64	1	–	–
TMS - CA	−0.70 (−2.64, 1.24)	20	1	–	–
VR - ACU	0.41 (−1.24, 2.06)	68	1	–	–
**BI**					
TMS - NOR	**11.22 (2.53, 19.90)**	260	3	95	<0.0001
tDCS - NOR	**10.46 (8.29, 12.64)**	195	4	48	0.12
VR - NOR	**5.52 (4.24, 6.80)**	274	6	0	0.69
CA - NOR	**5.44 (2.78, 8.11)**	238	4	41	0.17
ACU - NOR	**9.86 (6.22, 13.50)**	563	7	87	<0.0001
VR - ACU	1.94 (−0.57, 4.45)	68	1	–	–

TMS, Transcranial Magnetic Stimulation; VR, Virtual Reality Exposure Therapy; CA, Computer-assisted cognitive rehabilitation; tDCS, Transcranial Direct Current Stimulation; Acu, Acupuncture; NOR, Normal rehabilitation (including conventional rehabilitation and routine cognition training). Bold values means *p* < 0.05.

### Network meta-analysis

Network meta-analyses in the consistency model were conducted in the Bayesian framework to assess the efficacy of MOCA, MMSE, and BI, respectively. As shown in Supplementary Table 3, for each outcome, the PSRF value was equal to 1, indicating that the model had converged and that the findings were relatively stable.

**Figure 3:**
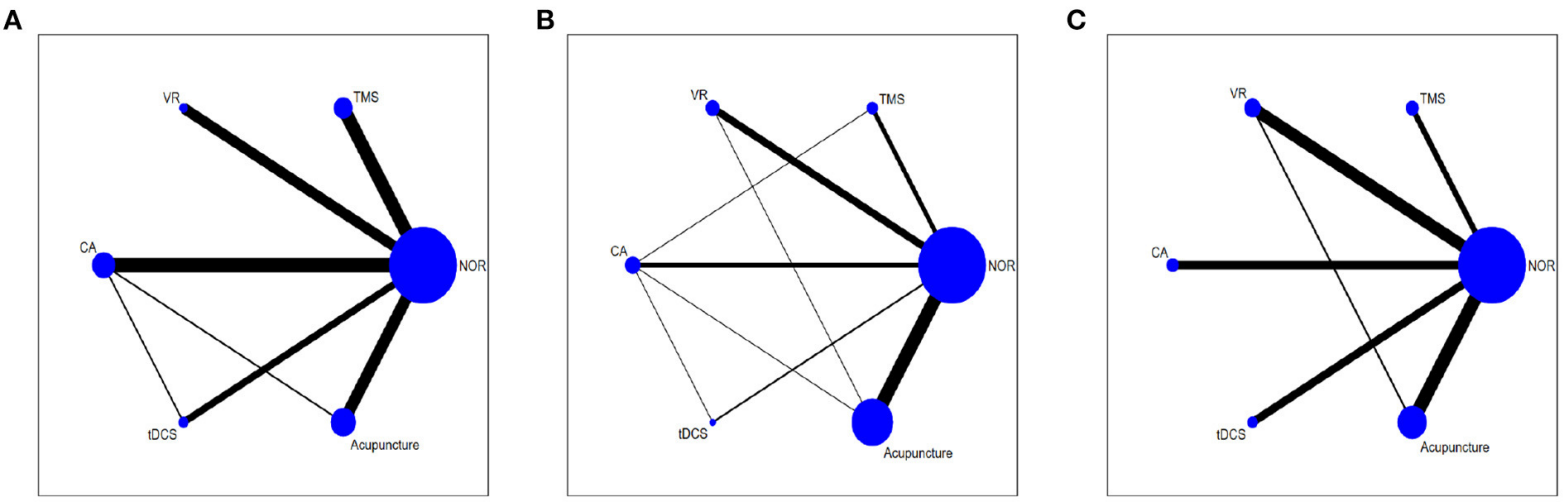
Network meta-analysis diagrams of eligible comparisons. **(A)** MOCA, **(B)** MMSE, **(C)** BI. Width of the lines is proportional to the number of trial. Size of every circle is proportional to the number of randomly assigned participants (sample size). TMS, Transcranial Magnetic Stimulation; VR, Virtual Reality Exposure Therapy; CA, Computer-assisted cognitive rehabilitation; tDCS, Transcranial Direct Current Stimulation; Acu, Acupuncture; NOR, Normal rehabilitation (including conventional rehabilitation and routine cognition training).

**Figure 4:**
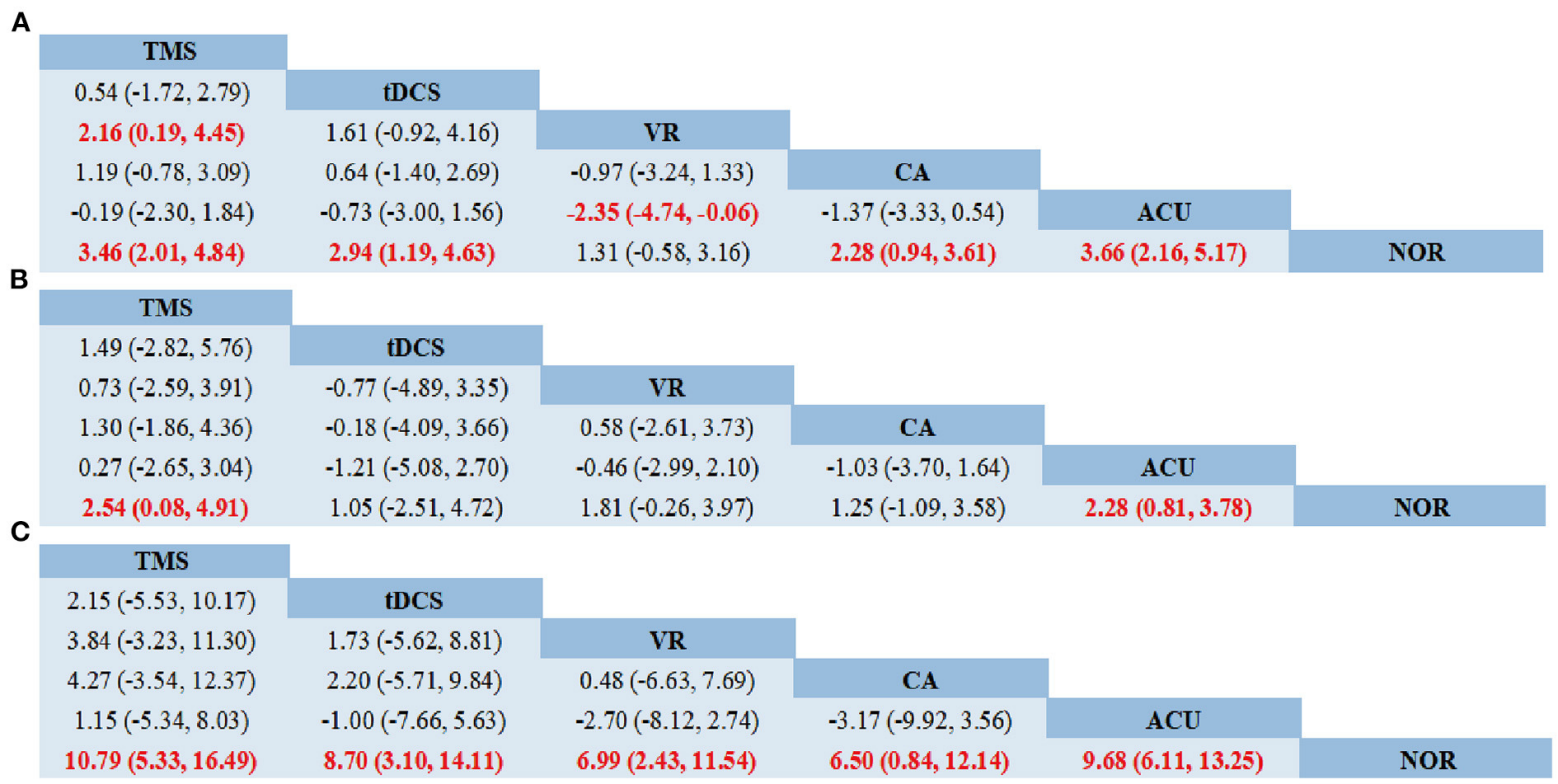
Network meta-analysis of head-to-head comparisons. **(A)** MOCA, **(B)** MMSE, **(C)** BI. Data are MD (95% CI) in the column-defining treatment compared with the row-defining treatment. Significant results are highlighted in red and bold. TMS, Transcranial Magnetic Stimulation; VR, Virtual Reality Exposure Therapy; CA, Computer-assisted cognitive rehabilitation; tDCS, Transcranial Direct Current Stimulation; Acu, Acupuncture; NOR, Normal rehabilitation.

**Figure 5:**
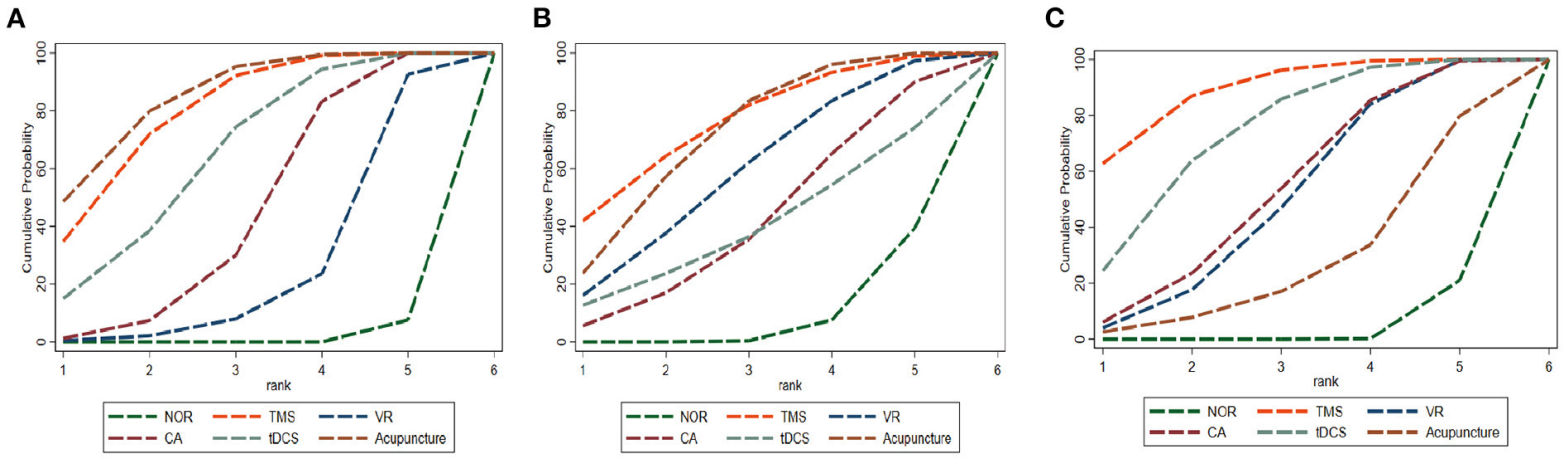
Cumulative probability ranking curve of different interventions. **(A)** MOCA, **(B)** MMSE, **(C)** BI. The vertical axis represents cumulative probabilities, while the horizontal axis represents ranks. TMS, Transcranial Magnetic Stimulation; VR, Virtual Reality Exposure Therapy; CA, Computer-assisted cognitive rehabilitation; tDCS, Transcranial Direct Current Stimulation; Acu, Acupuncture; NOR, Normal treatment (including conventional rehabilitation and routine cognition training).

As shown in network diagrams (Figure 3A), MoCA data were available from 33 studies that included 2,316 patients, of whom 1,102 in the NOR group, 293 in TMS, 109 in VR, 318 in CA, 138 in tDCS, and 356 in Acupuncture. The pooled MOCA data indicated that TMS (MD = 3.46, 95% CI: 2.01–4.84), tDCS (MD = 2.94, 95% CI: 1.19–4.63), CA (MD = 2.28, 95% CI: 0.94–3.61) and Acupuncture (MD = 3.66, 95% CI: 2.16–5.17) were more beneficial in patients compared with that of NOR. In addition, TMS and Acupuncture are better than VR when comparing the efficacy of the various therapies (Figure 4A). Based on the pooled data, the best therapies for MOCA were ranked as follows: Acupuncture, TMS, tDCS, CA, VR, and NOR (Figure 5A). The best SUCRA value for Acupuncture was 84.7%, which was close to that of TMS with a value of 79.7% (Supplementary Table 4).

In terms of MMSE, 35 studies with 2,573 patients were included in the network meta-analysis, of whom 1,191 were in the NOR group, 180 in TMS, 202 in VR, 216 in CA, 93 in tDCS, and 691 in Acupuncture (Figure 3B). The pooled data demonstrated a significant improvement for TMS (MD = 2.54, 95% CI: 0.08–4.91) and Acupuncture (MD = 2.28, 95% CI: 0.81–3.78) compared with that of NOR. Besides, significant differences were not observed between the other pairwise comparisons (Figure 4B). The best therapies for MMSE were ranked as TMS, Acupuncture, VR, tDCS, CA, and NOR (Figure 5B). And the best SUCRA value of TMS was 76.1%, which was close to that of Acupuncture with a SUCRA value of 72.1% (Supplementary Table 4).

For the outcome of BI, 23 studies with 1,496 patients were included in the network meta-analysis, of whom 726 were in the NOR group, 130 in TMS, 136 in VR, 119 in CA, 105 in tDCS, and 280 in Acupuncture (Figure 3C). The pooled data of all the 5 therapies demonstrated a significant improvement compared with that of NOR. However, when it comes to pairwise comparisons, no significant differences were found between the 5 therapies (Figure 4C). Despite this, SUCRA was performed, demonstrating that the best therapies for BI ranked as TMS, tDCS, Acupuncture, VR, CA, and NOR (Figure 5C). And the best SUCRA value of TMS was 89.1%, which was far higher than that of the others (Supplementary Table 4).

### Safety assessment

Adverse effects were reported only in 7 of the 55 included randomized controlled trials (Supplementary Table 5). The adverse effects reported were mild, such as dizziness and headache during TMS, itching, tingling and burning at the site of tDCS, and scalp hematoma after acupuncture. And there are no adverse effects reported in VR and CA.

### Publication bias

**Figure 6:**
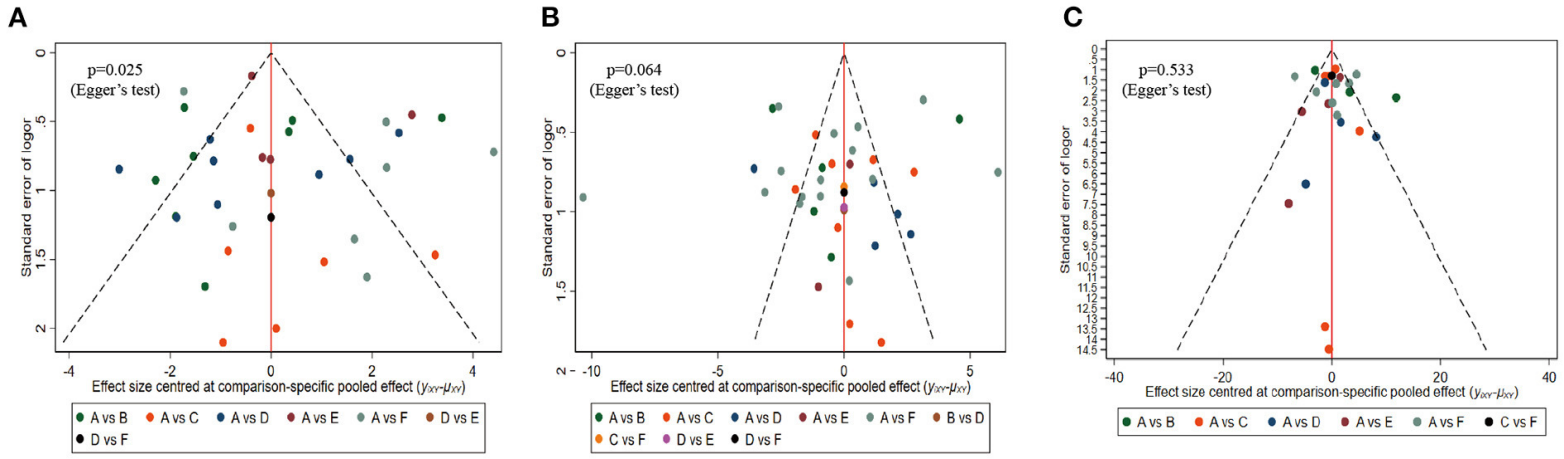
Comparison-adjusted funnel plots. **(A)** MOCA, **(B)** MMSE, **(C)** BI. Labels: A, NOR(Normal rehabilitation); B, TMS (Transcranial Direct Current Stimulation); C, VR (Virtual Reality Exposure Therapy); D, CA (Computer-assisted cognitive rehabilitation); E, tDCS (Transcranial Direct Current Stimulation); F, Acupuncture.

Comparison-adjusted funnel plots and Egger's test were performed to evaluate publication bias and small-study effects for MoCA, MMSE, and BI, respectively. Both the MMSE (Egger's test *p* = 0.064) and BI (Egger's test *p* = 0.533) comparison-adjusted funnel plots were rather symmetric, indicating that little publication bias likely occurred (Figures 6B,C). However, the MoCA (Egger's test *p* = 0.025) funnel plot was not well symmetrical and suggested a publication bias (Figure 6A).

### Transitivity, heterogeneity, and inconsistency assessment

Variables about patients known to affect how well a therapy works, such as age, percentage of male participants, sample size, publication year, percentage of ischemic stroke, education years, time post-stroke, course of treatment, and baseline indicators, were evaluated and visualized using box plots to assessed the transitivity assumption. As shown in Supplementary Figure 4, these characteristics across comparisons were relatively similar. The results of the test for inconsistency derived from the node-splitting model indicated that there was no significant difference in any of the comparisons across any of the outcomes, with the exception of the comparison of NOR vs. VR in BI (Supplementary Table 6). Similarly, when looking at the loop-specific inconsistency test, every loop included a value of 0, suggesting that no major contradiction was observed, with the exception of the NOR-VR-Acupuncture comparison in BI (Supplementary Figure 5). We then examined the goodness of fit between the inconsistency model and the consistency model to ensure there was no inconsistency at the treatment level. The DIC of the consistency model was 173.57 for MOCA, 188.21 for MMSE, and 227.48 for BI, which was similar to the DIC of the inconsistency model (174.11, 188.45, and 227.70, respectively), suggesting no evidence of inconsistency was found in the network. Low heterogeneity was found across most comparisons for all three outcomes, as measured by the prediction interval (Supplementary Figure 6). For comparisons with high heterogeneity, sensitivity analyses were performed and no studies that significantly increase heterogeneity was found (Supplementary Figures 7–9).

### GRADE evaluation on the quality of evidence

According to GRADE, the quality of the evidence is in the range of very low and moderate. In terms of TMS vs. ACU, the quality was moderate for MoCA, low for MMSE and BI. As for TMS vs. tDCS, the quality was low for BI. The details are shown in Supplementary Table 7.

## Discussion

According to the “Global Stroke Fact Sheet 2022” (10) published by the World Stroke Organization (WSO), reporting that stroke remains the second leading cause of death and the third leading cause of death and disability combined in the world. Although the development of effective acute treatments has resulted in global trends showing improvement in stroke outcomes (24), PSCI remains highly prevalent (25, 26) and associated closely with disability, dependency, and morbidity (6, 27), posing a major burden to patients, caregivers, and health care systems (8, 9). Thus, viable treatments are needed critically to help slow or stop the progression of PSCI. Unfortunately, there is no pharmacological treatment approved for PSCI, and prospective pharmaceutical medicines have yet to show significant efficacy in decreasing or preventing cognitive deterioration following a stroke (13, 28). Non-pharmacological interventions such as TMS, tDCS, VR, CA, and Acupuncture (16–20) have shown promise in several studies. However, there is continued uncertainty on the benefits due to methodological limitations that exist in most meta-analyses above, such as the unclear definition of PSCI, mixing of controlled groups, and combination of interventions in different groups. Besides, neither do we know whether there is a difference in efficacy among the non-pharmacological interventions mentioned above.

In this study, we conducted a Bayesian statistics NMA of 5 potential non-pharmacological therapies for PSCI patients. By comparing and ranking the treatments' curative effects on various outcomes, we were able to identify the treatment strategy that was most widely regarded as effective. In order to make the results more reliable, the participants in eligibility studies were limited to PSCI patients, the control interventions were limited to conventional rehabilitation combined with cognition training, and the 5 non-pharmacological interventions should not be applied in combination. Finally, two important findings have been obtained. Firstly, compared with the NOR, all five therapies had positive effects on some outcomes more or less. Secondly, TMS and Acupuncture are superior to NOR in all outcome indicators, with TMS being by far the most effective method for the improvement of MMSE and BI, and improvements in MoCA are most strongly associated with Acupuncture.

For the treatment of cerebral dysfunction brought on by a variety of disorders, TMS has shown to be an effective, painless, and non-invasive method of activating or modulating cortical targets in the central nervous system (CNS) (29, 30). Motor weakness, aphasia, and dysphagia have all been shown to improve with TMS treatment in clinical studies for individuals recovering from a stroke (31, 32). Furthermore, it has been recommended as “level A evidence” to use in the neurorehabilitation after motor stroke by the evidence-based guidelines (33). Evidence from the animal study suggests that the neuroprotective and pro-cognitive effects of TMS may exert by enhancing neurogenesis and activating BDNF/TrkB signaling pathway. A prospective pilot study conducted recently demonstrate that the scores of several cognitive evaluations increased after completion of the TMS session (34), which is similar to the results of pooled data in our study. However, it is worth noting that the stimulus parameters for TMS of the studies included in our network meta-analysis were not entirely consistent and subgroup analyses were not performed due to the limited literature, which may affect the reliability of the results to some extent.

Acupuncture, a well-known alternative treatment of traditional Chinese medicine with advantages of safety, reliability, and easy operation have been broadly applied to post-stroke patients. The positive effectiveness and safety of acupuncture in PSCI have been evaluated in a meta-analysis conducted recently (20). Studies in rats demonstrate that the improvement of the cognitive function performed by acupuncture may be associated with suppression of NF-κB-p53 activation and oxidative stress (35). Although acupuncture has been applied widely and a large number of articles have been published, just as the large number of articles related to acupuncture included in our network meta-analysis. However, we found that acupuncture ranked first only in terms of the probability of improving MoCA scores, with a tiny advantage compared with TMS. This may be related to the slow onset of acupuncture, and the evaluation time points of most studies included in our network meta-analysis in this study were at 4 weeks. In addition, the data from different types of acupuncture were pooled in the study, which may skew the results to some extent. However, more in-depth comparative studies are needed to verify this.

As shown in our study, tDCS and CA were effective only in improving MoCA scores but had no significant effect in improving MMSE, which may be related to the different characteristics between MoCA and MMSE scales. Studies have shown that compared with MMSE, MoCA is more sensitive to recognizing mild cognitive impairment, while MMSE is more suitable for the diagnosis of moderate to severe cognitive impairment (36, 37). In other words, MoCA is more likely to identify mild changes in cognitive function. This also implies, to some extent, that CA and tDCS are less effective in improving cognitive function in PSCI patients.

Furthermore, we were surprised to find that VR did little in improving MoCA and MMSE scores. Virtual reality (VR), a relatively new practical technology developed in the 20th century, allows for the seamless integration of training tasks into a simulated environment (such as a home, sports training facility, or social setting). This creates a more realistic, intuitive, and interactive feedback environment (38–40). Which is regarded as a conducive way of improving the neuroplasticity of the brain (41). However, the effectiveness of VR in improving global cognitive function in PSCI patients remains uncertain, just as demonstrated by several meta-analyses (41, 42). This may be related to the fact that current VR rehabilitation content is more focused on various immersive games that require more physical mobilization to cooperate. Additional factors, such as specific rehabilitation content of VR and the estimation of different dimensions of cognitive function should be taken into consideration in future studies, to get a more reliable and instructive result.

### Limitations

Our research has a number of drawbacks. First, the majority of the research included was conducted in China, which may have introduced bias and made the overall findings less compelling. Second, several of the RCTs included in the present study contained samples with < 30 people in each group, which raises concerns about the robustness of the findings. Fortunately, our network meta-analysis did not reveal any glaring inconsistencies or heterogeneities. Third, the study did not evaluate the scores of various dimensions of cognitive function, which may underestimate the effectiveness of some interventions. Finally, some baseline data related closely to cognitive function, such as volume and location of cerebral infarction, were not fully collected, which may reduce the credibility of the results. Fortunately, other important baseline data such as age, years of education, course of duration, etc., were collected and compared, and no significant differences were found.

## Conclusion

The results of this study provide some evidence that the 5 included therapies have positive effects for cognitive function on certain outcomes more or less. TMS may be the preferred therapy for improving MMSE and BI of PSCI patients, while acupuncture may be the preferred therapy in MOCA. CA and tDCS are also beneficial with less effective. The effects of VR are still waiting for more research to confirm.

## Data availability statement

The raw data supporting the conclusions of this article will be made available by the authors, without undue reservation.

## Author contributions

YZ conceived and designed the study and the guarantor of this study and accepts full responsibility for the work. ZL, LY, HQ, and XW independently assessed studies for possible inclusion and collected data. ZL, LY, HQ, XW, and CZ analyzed the data. ZL and LY wrote the manuscript. All authors contributed to the article and approved the submitted version.

## Funding

This work was supported by the grants of Hunan Provincial Health Commission Scientific Research Project (No.202114011882), Hunan Provincial Traditional Chinese Medicine Scientific Research Project (No.2020004), and Hunan Clinical Medical Technology Innovation Guidance Project (No.2020SK51401).

## Conflict of interest

The authors declare that the research was conducted in the absence of any commercial or financial relationships that could be construed as a potential conflict of interest.

## Publisher's note

All claims expressed in this article are solely those of the authors and do not necessarily represent those of their affiliated organizations, or those of the publisher, the editors and the reviewers. Any product that may be evaluated in this article, or claim that may be made by its manufacturer, is not guaranteed or endorsed by the publisher.

## Abbreviations

PSCI: Post-stroke cognitive impairment
TMS: Transcranial Magnetic Stimulation
tDCS: Transcranial Direct Current Stimulation
CA: Computer-assisted cognitive rehabilitation
VR: Virtual Reality Exposure Therapy
NOR: Normal rehabilitation
MMSE: Mini-Mental State Examination
MoCA: Montreal Cognitive Assessment Scale
NMA: Network Meta-analysis
RCTs: Randomized Controlled Trials
MD: Mean Differences
CI: Confidence Interval
PSRF: Potential Size Reduction Factor
SUCRA: Surface Under The Cumulative Ranking Area
DIC: Deviance Information Criterion.
