# Supplementary material for: Comparative efficacy of 5 non-pharmacological therapies for adults with post-stroke cognitive impairment: A Bayesian network analysis based on 55 randomized controlled trials
Source: Front Neurol. 2022 Sep 28;13:977518. doi: 10.3389/fneur.2022.977518 (PMC9554560; doi:10.3389/fneur.2022.977518)
Supplement: Supplementary file 1 [file Data_Sheet_1.PDF]

frontiers

Supplementary Material

1. Supplementary Tables

Supplementary Table 1. The Search Strategies

Pubmed(127)	
#1	("Stroke"[Mesh]) OR ("Brain Ischemia"[Mesh]) OR ("Cerebral Hemorrhage"[Mesh]) OR (Stroke*[Title/Abstract]) OR (Cerebrovascular Accident*[Title/Abstract]) OR (Brain Vascular Accident*[Title/Abstract]) OR (Apoplexy[Title/Abstract]) OR (Brain Infarct*[Title/Abstract]) OR (Cerebral Infarct*[Title/Abstract]) OR (Brain Stem Infarct*[Title/Abstract]) OR (Subcortical Infarction*[Title/Abstract]) OR (Brain Venous Infarction*[Title/Abstract]) OR (Cerebral Artery Stroke[Title/Abstract]) OR (Cerebral Artery Infarction[Title/Abstract]) OR (Cerebral Circulation Infarction[Title/Abstract]) OR (Circulation Brain Infarction[Title/Abstract]) OR (Choroidal Artery Infarction[Title/Abstract]) OR (CVA[Title/Abstract]) OR (CVAs[Title/Abstract]) OR (Brain Ischemia*[Title/Abstract]) OR (Ischemic Encephalopath*[Title/Abstract]) OR (Cerebral Ischemia*[Title/Abstract]) OR (Brain Hypoxia Ischemia*[Title/Abstract]) OR (Cerebral Anoxia Ischemia*[Title/Abstract]) OR (Cerebral Hemorrhage*[Title/Abstract]) OR (Cerebral Brain Hemorrhage*[Title/Abstract]) OR (Cerebral Parenchymal Hemorrhage*[Title/Abstract]) OR (Cerebrum Hemorrhage*[Title/Abstract]) OR (Intracerebral Hemorrhage*[Title/Abstract]) OR (Basal Ganglia Hemorrhage[Title/Abstract]) OR (Subarachnoid Hemorrhage*[Title/Abstract]) OR (Cerebral Hypertensive Hemorrhage*[Title/Abstract])
#2	(Cognition Disorders[MeSH Terms]) OR (Dementia, Vascular[MeSH Terms]) OR (Cognition Disorder*[Title/Abstract]) OR (Vascular Dementia*[Title/Abstract]) OR (Cognitive Dysfunction*[Title/Abstract]) OR (Cognitive Impairment*[Title/Abstract]) OR (Mild Neurocognitive Disorder*[Title/Abstract]) OR (Cognitive Decline*[Title/Abstract]) OR (Mental Deterioration*[Title/Abstract]) OR (cognitive defect[Title/Abstract]) OR (Multi-Infarct Dementia*[Title/Abstract]) OR (Lacunar Dementia*[Title/Abstract]) OR (Arteriosclerotic Dementia*[Title/Abstract]) OR (Chronic Progressive Subcortical Encephalopathy[Title/Abstract]) OR (Subcortical Leukoencephalopathies[Title/Abstract]) OR (cognitive disorder* after stroke[Title/Abstract]) OR (cognitive impairment* after stroke[Title/Abstract]) OR (cognitive Dysfunction* after stroke[Title/Abstract]) OR (Cognitive Decline* after stroke[Title/Abstract])
#3	(Transcranial Magnetic Stimulation[MeSH Terms]) OR (Transcranial Direct Current Stimulation[MeSH Terms]) OR (Acupuncture[MeSH Terms]) OR (Acupuncture Therapy[MeSH Terms]) OR (Electroacupuncture[MeSH Terms]) OR (Virtual Reality[MeSH Terms]) OR (Virtual Reality Exposure Therapy[MeSH Terms]) OR (Transcranial Magnetic Stimulation*[Title/Abstract]) OR (TMS[Title/Abstract]) OR (tDCS[Title/Abstract]) OR (Transcranial Direct Current Stimulation*[Title/Abstract]) OR (Cathodal Stimulation tDCS*[Title/Abstract]) OR (Transcranial Random Noise Stimulation[Title/Abstract]) OR (Transcranial Alternating Current Stimulation[Title/Abstract]) OR

	(Transcranial Electrical Stimulation*[Title/Abstract]) OR (Anodal Stimulation tDCS*[Title/Abstract]) OR (Pharmacopuncture[Title/Abstract]) OR (Acupuncture[Title/Abstract]) OR (Acupuncture Therapy[Title/Abstract]) OR (Acupuncture Treatment*[Title/Abstract]) OR (Pharmacoacupuncture Treatment[Title/Abstract]) OR (Pharmacoacupuncture Therapy[Title/Abstract]) OR (Acupotomy[Title/Abstract]) OR (Acupotomies[Title/Abstract]) OR (Electroacupuncture[Title/Abstract]) OR (Virtual Reality[Title/Abstract]) OR (VR[Title/Abstract]) OR (Virtual Reality Exposure Therapy[Title/Abstract]) OR (Virtual Reality Immersion Therapy[Title/Abstract]) OR (Virtual Reality Therapy[Title/Abstract]) OR (Virtual Reality Therapies[Title/Abstract]) OR (computer-assisted cognitive rehabilitation[Title/Abstract]) OR (((cognitive[Title/Abstract]) OR (cognition[Title/Abstract])) AND (computer*[Title/Abstract]))
#4	Randomized controlled trial[Publication Type] OR randomized[Title/Abstract] OR placebo[Title/Abstract]
#5	#1 AND #2 AND #3 AND #4
**Embase(292)**	
#1	'cerebrovascular accident'/exp OR 'brain hemorrhage'/exp OR 'brain infarction'/exp OR 'brain ischemia'/exp
#2	'Stroke':ab,ti OR 'Brain Ischemia':ab,ti OR 'Cerebral Hemorrhage':ab,ti OR 'Stroke*':ab,ti OR 'Cerebrovascular Accident*':ab,ti OR 'Brain Vascular Accident*':ab,ti OR 'Apoplexy':ab,ti OR 'Brain Infarct*':ab,ti OR 'Cerebral Infarct*':ab,ti OR 'Brain Stem Infarct*':ab,ti OR 'Subcortical Infarction*':ab,ti OR 'Brain Venous Infarction*':ab,ti OR 'Cerebral Artery Stroke':ab,ti OR 'Cerebral Artery Infarction':ab,ti OR 'Cerebral Circulation Infarction':ab,ti OR 'Circulation Brain Infarction':ab,ti OR 'Choroidal Artery Infarction':ab,ti OR 'CVA':ab,ti OR 'CVAs':ab,ti OR 'Brain Ischemia*':ab,ti OR 'Ischemic Encephalopath*':ab,ti OR 'Cerebral Ischemia*':ab,ti OR 'Brain Hypoxia Ischemia*':ab,ti OR 'Cerebral Anoxia Ischemia*':ab,ti OR 'Cerebral Hemorrhage*':ab,ti OR 'Cerebral Brain Hemorrhage*':ab,ti OR 'Cerebral Parenchymal Hemorrhage*':ab,ti OR 'Cerebrum Hemorrhage*':ab,ti OR 'Intracerebral Hemorrhage*':ab,ti OR 'Basal Ganglia Hemorrhage':ab,ti OR 'Subarachnoid Hemorrhage*':ab,ti OR 'Cerebral Hypertensive Hemorrhage*':ab,ti
#3	'cognitive defect'/exp
#4	'cognitive defect':ab,ti OR 'Cognition Disorder*':ab,ti OR 'Vascular Dementia*':ab,ti OR 'Cognitive Dysfunction*':ab,ti OR 'Cognitive Impairment*':ab,ti OR 'Mild Neurocognitive Disorder*':ab,ti OR 'Cognitive Decline*':ab,ti OR 'Mental Deterioration*':ab,ti OR 'Multi-Infarct Dementia*':ab,ti OR 'Lacunar Dementia*':ab,ti OR 'Arteriosclerotic Dementia*':ab,ti OR 'Chronic Progressive Subcortical Encephalopathy':ab,ti OR 'Subcortical Leukoencephalopathies':ab,ti OR 'cognitive disorder* after stroke':ab,ti OR 'cognitive impairment*

	after stroke':ab,ti OR 'cognitive Dysfunction* after stroke':ab,ti OR 'Cognitive Decline* after stroke':ab,ti
#5	'transcranial magnetic stimulation'/exp OR 'transcranial direct current stimulation'/exp OR 'acupuncture'/exp OR 'virtual reality'/exp OR 'virtual reality exposure therapy'/exp
#6	'transcranial magnetic stimulation':ab,ti OR 'transcranial direct current stimulation':ab,ti OR 'transcranial magnetic stimulation*':ab,ti OR 'tms':ab,ti OR 'tdcs':ab,ti OR 'transcranial direct current stimulation*':ab,ti OR 'cathodal stimulation tdcs*':ab,ti OR 'transcranial random noise stimulation':ab,ti OR 'transcranial alternating current stimulation':ab,ti OR 'transcranial electrical stimulation*':ab,ti OR 'anodal stimulation tdcs*':ab,ti OR 'pharmacopuncture':ab,ti OR 'acupuncture':ab,ti OR 'acupuncture therapy':ab,ti OR 'acupuncture treatment*':ab,ti OR 'pharmacoacupuncture treatment':ab,ti OR 'pharmacoacupuncture therapy':ab,ti OR 'acupotomy':ab,ti OR 'acupotomies':ab,ti OR 'electroacupuncture':ab,ti OR 'virtual reality':ab,ti OR 'vr':ab,ti OR 'virtual reality exposure therapy':ab,ti OR 'virtual reality immersion therapy':ab,ti OR 'virtual reality therapy':ab,ti OR 'virtual reality therapies':ab,ti OR 'computer-assisted cognitive rehabilitation':ab,ti OR (('cognitive':ab,ti OR 'cognition':ab,ti) AND 'computer*':ab,ti)
#7	'randomized controlled trial'/exp OR randomized:ab,ti OR placebo:ab,ti
#8	(#1 OR #2) AND (#3 OR #4) AND (#5 OR #6) AND #7
**Cochrane(354)**	
#1	MeSH descriptor: [Stroke] explode all trees
#2	MeSH descriptor: [Brain Ischemia] explode all trees
#3	MeSH descriptor: [Cerebral Hemorrhage] explode all trees
#4	(Stroke*):ti,ab,kw OR (Brain Ischemia):ti,ab,kw OR (Cerebral Hemorrhage):ti,ab,kw OR (Cerebrovascular Accident*):ti,ab,kw OR (Brain Vascular Accident*):ti,ab,kw OR (Apoplexy):ti,ab,kw OR (Brain Infarct*):ti,ab,kw OR (Cerebral Infarct*):ti,ab,kw OR (Brain Stem Infarct*):ti,ab,kw OR (Subcortical Infarction*):ti,ab,kw OR (Brain Venous Infarction*):ti,ab,kw OR (Cerebral Artery Stroke):ti,ab,kw OR (Cerebral Artery Infarction):ti,ab,kw OR (Cerebral Circulation Infarction):ti,ab,kw OR (Circulation Brain Infarction):ti,ab,kw OR (Choroidal Artery Infarction):ti,ab,kw OR (CVA):ti,ab,kw OR (CVAs):ti,ab,kw OR (Brain Ischemia*):ti,ab,kw OR (Ischemic Encephalopath*):ti,ab,kw OR (Cerebral Ischemia*):ti,ab,kw OR (Brain Hypoxia Ischemia*):ti,ab,kw OR (Cerebral Anoxia Ischemia*):ti,ab,kw OR (Cerebral Hemorrhage*):ti,ab,kw OR (Cerebral Brain Hemorrhage*):ti,ab,kw OR (Cerebral Parenchymal Hemorrhage*):ti,ab,kw OR (Cerebrum Hemorrhage*):ti,ab,kw OR (Intracerebral Hemorrhage*):ti,ab,kw OR (Basal Ganglia

	Hemorrhage):ti,ab,kw OR (Subarachnoid Hemorrhage*):ti,ab,kw OR (Cerebral Hypertensive Hemorrhage*):ti,ab,kw
#5	MeSH descriptor: [Cognition Disorders] explode all trees
#6	MeSH descriptor: [Dementia, Vascular] explode all trees
#7	(Cognition Disorder*):ti,ab,kw OR (Vascular Dementia*):ti,ab,kw OR (Cognitive Dysfunction*):ti,ab,kw OR (Cognitive Impairment*):ti,ab,kw OR (Mild Neurocognitive Disorder*):ti,ab,kw OR (Cognitive Decline*):ti,ab,kw OR (Mental Deterioration*):ti,ab,kw OR (cognitive defect):ti,ab,kw OR (Multi-Infarct Dementia*):ti,ab,kw OR (Lacunar Dementia*):ti,ab,kw OR (Arteriosclerotic Dementia*):ti,ab,kw OR (Chronic Progressive Subcortical Encephalopathy):ti,ab,kw OR (Subcortical Leukoencephalopathies):ti,ab,kw OR (cognitive disorder* after stroke):ti,ab,kw OR (cognitive impairment* after stroke):ti,ab,kw OR (cognitive Dysfunction* after stroke):ti,ab,kw OR (Cognitive Decline* after stroke):ti,ab,kw
#8	MeSH descriptor: [Transcranial Magnetic Stimulation] explode all trees
#9	MeSH descriptor: [Transcranial Direct Current Stimulation] explode all trees
#10	MeSH descriptor: [Acupuncture] explode all trees
#11	MeSH descriptor: [Acupuncture Therapy] explode all trees
#12	MeSH descriptor: [Electroacupuncture] explode all trees
#13	MeSH descriptor: [Virtual Reality] explode all trees
#14	MeSH descriptor: [Virtual Reality Exposure Therapy] explode all trees
#15	(Transcranial Magnetic Stimulation*):ti,ab,kw OR (TMS):ti,ab,kw OR (tDCS):ti,ab,kw OR (Transcranial Direct Current Stimulation*):ti,ab,kw OR (Cathodal Stimulation tDCS*):ti,ab,kw OR (Transcranial Random Noise Stimulation):ti,ab,kw OR (Transcranial Alternating Current Stimulation):ti,ab,kw OR (Transcranial Electrical Stimulation*):ti,ab,kw OR (Anodal Stimulation tDCS*):ti,ab,kw OR (Pharmacopuncture):ti,ab,kw OR (Acupuncture):ti,ab,kw OR (Acupuncture Therapy):ti,ab,kw OR (Acupuncture Treatment*):ti,ab,kw OR (Pharmacoacupuncture Treatment):ti,ab,kw OR (Pharmacoacupuncture Therapy):ti,ab,kw OR (Acupotomy):ti,ab,kw OR (Acupotomies):ti,ab,kw OR (Electroacupuncture):ti,ab,kw OR (Virtual Reality):ti,ab,kw OR (VR):ti,ab,kw OR (Virtual Reality Exposure Therapy):ti,ab,kw OR (Virtual Reality Immersion Therapy):ti,ab,kw OR (Virtual Reality Therapy):ti,ab,kw OR (Virtual Reality Therapies):ti,ab,kw OR (computer-assisted cognitive rehabilitation):ti,ab,kw OR (((cognitive):ti,ab,kw OR

	(cognition):ti,ab,kw) AND (computer*):ti,ab,kw)
#16	(Randomized controlled trial):pt OR (randomized):ti,ab,kw OR (placebo):ti,ab,kw
#17	(#1 OR #2 OR #3 OR #4) AND (#5 OR #6 OR #7) AND (#8 OR #9 OR #10 OR #11 OR #12 OR #13 OR #14 OR #15) AND #16
**Web of science(203)**	
#1	TS=(Stroke) OR TS=(Brain Ischemia) OR TS=(Cerebral Hemorrhage) OR TS=(Stroke*) OR TS=(Cerebrovascular Accident*) OR TS=(Brain Vascular Accident*) OR TS=(Apoplexy) OR TS=(Brain Infarct*) OR TS=(Cerebral Infarct*) OR TS=(Brain Stem Infarct*) OR TS=(Subcortical Infarction*) OR TS=(Brain Venous Infarction*) OR TS=(Cerebral Artery Stroke) OR TS=(Cerebral Artery Infarction) OR TS=(Cerebral Circulation Infarction) OR TS=(Circulation Brain Infarction) OR TS=(Choroidal Artery Infarction) OR TS=(CVA) OR TS=(CVAs) OR TS=(Brain Ischemia*) OR TS=(Ischemic Encephalopath*) OR TS=(Cerebral Ischemia*) OR TS=(Brain Hypoxia Ischemia*) OR TS=(Cerebral Anoxia Ischemia*) OR TS=(Cerebral Hemorrhage*) OR TS=(Cerebral Brain Hemorrhage*) OR TS=(Cerebral Parenchymal Hemorrhage*) OR TS=(Cerebrum Hemorrhage*) OR TS=(Intracerebral Hemorrhage*) OR TS=(Basal Ganglia Hemorrhage) OR TS=(Subarachnoid Hemorrhage*) OR TS=(Cerebral Hypertensive Hemorrhage*)
#2	TS=(Cognition Disorders) OR TS=(Cognition Disorder*) OR TS=(Vascular Dementia*) OR TS=(Cognitive Dysfunction*) OR TS=(Cognitive Impairment*) OR TS=(Mild Neurocognitive Disorder*) OR TS=(Cognitive Decline*) OR TS=(Mental Deterioration*) OR TS=(cognitive defect) OR TS=(Multi-Infarct Dementia*) OR TS=(Lacunar Dementia*) OR TS=(Arteriosclerotic Dementia*) OR TS=(Chronic Progressive Subcortical Encephalopathy) OR TS=(Subcortical Leukoencephalopathies) OR TS=(cognitive disorder* after stroke) OR TS=(cognitive impairment* after stroke) OR TS=(cognitive Dysfunction* after stroke) OR TS=(Cognitive Decline* after stroke)
#3	TS=(Transcranial Magnetic Stimulation) OR TS=(Transcranial Direct Current Stimulation) OR TS=(Acupuncture) OR TS=(Acupuncture Therapy) OR TS=(Electroacupuncture) OR TS=(Virtual Reality) OR TS=(Virtual Reality Exposure Therapy) OR TS=(Transcranial Magnetic Stimulation*) OR TS=(TMS) OR TS=(tDCS) OR TS=(Transcranial Direct Current Stimulation*) OR TS=(Cathodal Stimulation tDCS*) OR TS=(Transcranial Random Noise Stimulation) OR TS=(Transcranial Alternating Current Stimulation) OR TS=(Transcranial Electrical Stimulation*) OR TS=(Anodal Stimulation tDCS*) OR TS=(Pharmacopuncture) OR TS=(Acupuncture) OR TS=(Acupuncture Therapy) OR TS=(Acupuncture Treatment*) OR TS=(Pharmacoacupuncture Treatment) OR TS=(Pharmacoacupuncture Therapy) OR TS=(Acupotomy) OR TS=(Acupotomies) OR TS=(Electroacupuncture) OR TS=(Virtual Reality) OR TS=(VR) OR TS=(Virtual Reality

	Exposure Therapy) OR TS=(Virtual Reality Immersion Therapy) OR TS=(Virtual Reality Therapy) OR TS=(Virtual Reality Therapies) OR TS=(computer-assisted cognitive rehabilitation) OR ((TS=(cognitive) OR TS=(cognition)) AND TS=(computer*))
#4	TS=(Randomized controlled trial) OR TS=(randomized) OR TS=(placebo)
#5	#1 AND #2 AND #3 AND #4
**SinoMed(490)**	
#1	"卒中"[加权:扩展] OR "中风"[加权:扩展] OR "脑缺血"[加权:扩展] OR "颅内出血"[加权:扩展]
#2	"中风"[常用字段:智能] OR "风痱"[常用字段:智能] OR "卒中"[常用字段:智能] OR "脑卒中"[常用字段:智能] OR "脑中风"[常用字段:智能] OR "脑血管意外"[常用字段:智能] OR "脑血管中风"[常用字段:智能] OR "脑梗死"[常用字段:智能] OR "脑静脉梗塞"[常用字段:智能] OR "大脑梗死"[常用字段:智能] OR "脑栓塞"[常用字段:智能] OR "脑梗塞"[常用字段:智能] OR "皮层下梗塞"[常用字段:智能] OR "脑干梗死"[常用字段:智能] OR "脑干梗死"[常用字段:智能] OR "脑缺血"[常用字段:智能] OR "脑缺血症"[常用字段:智能] OR "缺血性脑病"[常用字段:智能] OR "脑干缺血"[常用字段:智能] OR "脑出血"[常用字段:智能] OR "脑实质出血"[常用字段:智能] OR "脑内出血"[常用字段:智能] OR "脑内出血"[常用字段:智能] OR "蛛网膜下腔出血"[常用字段:智能] OR "颅内出血"[常用字段:智能]
#3	"认知障碍"[不加权:扩展] OR "痴呆，血管性"[不加权:扩展]
#4	"认知障碍"[常用字段:智能] OR "认知功能障碍"[常用字段:智能] OR "认知减退"[常用字段:智能] OR "认知损害"[常用字段:智能] OR "精神衰退"[常用字段:智能] OR "轻度认知障碍"[常用字段:智能] OR "轻度神经认知障碍"[常用字段:智能] OR "痴呆"[常用字段:智能] OR "血管性痴呆"[常用字段:智能] OR "动脉硬化性痴呆"[常用字段:智能] OR "腔隙性痴呆"[常用字段:智能] OR "多发性梗塞性痴呆"[常用字段:智能]
#5	"经颅磁刺激"[不加权:扩展] OR "经颅直流电刺激"[不加权:扩展] OR "虚拟现实暴露疗法"[不加权:扩展] OR "针灸疗法"[不加权:扩展] OR "针刺疗法"[不加权:扩展]
#6	"经颅磁刺激"[常用字段:智能] OR "TMS"[常用字段:智能] OR "经颅直流电刺激"[常用字段:智能] OR "TDCS"[常用字段:智能] OR "虚拟现实暴露疗法"[常用字段:智能] OR "虚拟现实"[常用字段:智能] OR "VR"[常用字段:智能] OR "针灸疗法"[常用字段:智能] OR "针刺疗法"[常用字段:智能] OR "针灸"[常用字段:智能] OR "针刺"[常用字段:智能] OR "头针"[常用字段:智能] OR "电针"[常用字段:智能] OR (("认知训练"[常用字段:智能] OR "认知康复"[常用字段:智能]) AND "计算机"[常用字段:智能])
#7	”随机对照试验”[文献类型])
#8	(#1 OR #2) AND (#3 OR #4) AND (#5 OR #6) AND #7

**CNKI(773)**	
	TKA=(中风 + 风痱 + 卒中 + 脑卒中 + 脑中风 + 脑血管意外 + 脑血管中风 + 脑梗死 + 脑静脉梗塞 + 大脑梗死 + 脑栓塞 + 脑梗塞 + 皮层下梗塞 + 脑干梗死 + 脑干梗死 + 脑缺血 + 脑缺血症 + 缺血性脑病 + 脑干缺血 + 脑出血 + 脑实质出血 + 脑内出血 + 脑内出血 + 蛛网膜下腔出血 + 颅内出血) AND TKA=(认知障碍 + 认知功能障碍 + 认知减退 + 认知损害 + 精神衰退 + 轻度认知障碍 + 轻度神经认知障碍 + 痴呆 + 血管性痴呆 + 动脉硬化性痴呆 + 腔隙性痴呆 + 多发性梗塞性痴呆) AND TKA=(经颅磁刺激 + TMS + 经颅直流电刺激 + TDCS + 虚拟现实暴露疗法 + 虚拟现实 + VR + 针灸疗法 + 针刺疗法 + 针灸 + 针刺 + 头针 + 电针 + ((认知训练 + 认知康复) AND 计算机)) AND TKA=(随机对照试验 + 随机对照 + 随机)
**WanFang(821)**	
	主题：(中风 or 风痱 or 卒中 or 脑卒中 or 脑中风 or 脑血管意外 or 脑血管中风 or 脑梗死 or 脑静脉梗塞 or 大脑梗死 or 脑栓塞 or 脑梗塞 or 皮层下梗塞 or 脑干梗死 or 脑干梗死 or 脑缺血 or 脑缺血症 or 缺血性脑病 or 脑干缺血 or 脑出血 or 脑实质出血 or 脑内出血 or 脑内出血 or 蛛网膜下腔出血 or 颅内出血) AND 主题：(认知障碍 or 认知功能障碍 or 认知减退 or 认知损害 or 精神衰退 or 轻度认知障碍 or 轻度神经认知障碍 or 痴呆 or 血管性痴呆 or 动脉硬化性痴呆 or 腔隙性痴呆 or 多发性梗塞性痴呆) AND 题名或关键词：(经颅磁刺激 or TMS or 经颅直流电刺激 or TDCS or 虚拟现实暴露疗法 or 虚拟现实 or VR or 针灸疗法 or 针刺疗法 or 针灸 or 针刺 or 头针 or 电针 or ((认知训练 or 认知康复) and 计算机)) AND 主题:(随机对照试验 or 随机对照 or 随机))
**VIP(507)**	
	(M=(中风 or 风痱 or 卒中 or 脑卒中 or 脑中风 or 脑血管意外 or 脑血管中风 or 脑梗死 or 脑静脉梗塞 or 大脑梗死 or 脑栓塞 or 脑梗塞 or 皮层下梗塞 or 脑干梗死 or 脑干梗死 or 脑缺血 or 脑缺血症 or 缺血性脑病 or 脑干缺血 or 脑出血 or 脑实质出血 or 脑内出血 or 脑内出血 or 蛛网膜下腔出血 or 颅内出血) OR R=(中风 or 风痱 or 卒中 or 脑卒中 or 脑中风 or 脑血管意外 or 脑血管中风 or 脑梗死 or 脑静脉梗塞 or 大脑梗死 or 脑栓塞 or 脑梗塞 or 皮层下梗塞 or 脑干梗死 or 脑干梗死 or 脑缺血 or 脑缺血症 or 缺血性脑病 or 脑干缺血 or 脑出血 or 脑实质出血 or 脑内出血 or 脑内出血 or 蛛网膜下腔出血 or 颅内出血)) AND (M=(认知障碍 or 认知功能障碍 or 认知减退 or 认知损害 or 精神衰退 or 轻度认知障碍 or 轻度神经认知障碍 or 痴呆 or 血管性痴呆 or 动脉硬化性痴呆 or 腔隙性痴呆 or 多发性梗塞性痴呆) OR R=(认知障碍 or 认知功能障碍 or 认知减退 or 认知损害 or 精神衰退 or 轻度认知障碍 or 轻度神经认知障碍 or 痴呆 or 血管性痴呆 or 动脉硬化性痴呆 or 腔隙性痴呆 or 多发性梗塞性痴呆)) AND (M=(经颅磁刺激 or TMS or 经颅直流电刺激 or TDCS or 虚拟现实暴露疗法 or 虚拟现实 or VR or 针灸疗法 or 针刺疗法 or 针灸 or 针刺 or 头针 or 电针 or ((认知训练 or 认知康复) and 计算机))) AND (M=(随机对照试验 or 随机对照 or 随机) OR R=(随机对照试验 or 随机对照 or 随机))

Supplementary Table 2. Characteristics of 55 RCTs included in the network meta-analysis

Study	Country	Therapy	Sample size	Age/mean(SD)	Gender (Male/Female)	Course of treatment(weeks)	Outcomes
Huang CY 2021[1]	China	TMS-NOR	12-12	63.87(6.31)-61.48(9.08)	12/0-10/2	4	MOCA
Ma J 2021[2]	China	TMS-NOR	37-38	60.95(7.92)-58.84(10.89)	25/12-22/16	4	MOCA
Wang X 2021[3]	China	TMS-NOR	60-60	64.91(4.15)-65.30(4.81)	28/32-36/24	4	MOCA, MMSE
Zhang J 2021[4]	China	TMS-NOR	21-22	60.67(9.53)-58.95(7.88)	15/6-14/8	4	MOCA, MMSE
Liu Y 2020[5]	China	TMS-NOR	29-29	58.55(6.24)-57.69(7.25)	10/19-16/13	4	MMSE
Cheng ZY 2019[6]	China	TMS-NOR	70-70	57.1(5)-56.8(4.8)	37/33-39/31	4	MOCA, MBI
Zheng J 2017[7]	China	TMS-NOR	30-30	58.8(13.5)-61.97(11.39	14/16-17/13	6	MOCA, MMSE, MBI
Hu LM 2016[8]	China	TMS-NOR	30-30	57.5(13.3)-56.2(10.9)	14/16-15/15	4	MOCA, MMSE, MBI
Xu QQ 2018[9]	China	TMS-NOR	33-35	48.3(4.2)-47.9(6.3)	23/10-24/11	4	MOCA
Faria AL 2020[10]	Portugal	VR-NOR	14-18	59.14(11.81)-65.00(6.20)	5/9-11/7	8	MOCA
Ren YG 2020[11]	China	VR-NOR	40-40	58.63(4.76)-59.03(4.75)	24/16-22/18	4	MOCA, MBI
Guo JQ 2020[12]	China	VR-NOR	25-25	60.23(2.33)-60.56(2.24)	15/10-16/9	4	MMSE, MBI
Xue H 2020[13]	China	VR-NOR	50-50	67.2(9.4)-67.3(9.4)	26/24-29/21	NG	MMSE
Oh Y 2019[14]	Korea	VR-NOR	17-14	57.4(12.2)-52.6(10.7)	12/5-9/5	4	MOCA, MMSE
Rogers JM 2019[15]	Australian	VR-NOR	10-10	64.3(17.4)-64.6(12.0)	4/6-5/6	4	MOCA
Fu KJ 2019[16]	China	VR-NOR	18-18	57.3(5.9)-56.9(4.3)	11/7-10/8	4	MMSE
Xiao X 2019[17]	China	VR-NOR	16-18	67.74(9.35)-70.59(10.67)	9/7-12/6	4	MOCA, MMSE, MBI
Faria AL 2018[18]	Portugal	VR-NOR	12-12	57.1(11.0)-68.9(9.8)	8/4-7/5	4	MOCA, MBI

Hu YQ 2018[19]	China	VR-NOR	33-33	71.1(2.9)-70.3(2.2)	27/6-25/8	4	MMSE
Faria AL 2016[20]	Portugal	VR-NOR	9-9	58(17.04)-53(11.11)	4/5-4/5	4	MMSE, MBI
Zhang L 2020[21]	China	CA-NOR	37-37	71.5(5.3)-71.6(5.7)	20/17-21/16	6	MMSE
Luo XJ 2020[22]	China	CA-NOR	30-30	NG	NG	3	MOCA
Liu RF 2019[23]	China	CA-NOR	15-15	58.33(8.19)-54.40(9.23)	9/6-7/8	12	MOCA, MBI
Chen JJ 2019[24]	China	CA-NOR	24-24	60.46(7.51)-59.96(7.68)	14/10-15/9	6	MOCA, MBI
Lu CH 2019[25]	China	CA-NOR	50-50	70.56(5.65)-71.24(5.70)	21/29-22/28	8	MOCA, MBI
Liu X 2018[26]	China	CA-NOR	62-66	61.50(12.34)-62.35(10.34)	40/22-46/20	4	MOCA
De Luca 2018[27]	Portugal	CA-NOR	20-15	43.9(16.6)-42.1(17.7)	11/9-7/8	8	MMSE
Xiao TS 2018[28]	China	CA-NOR	30-30	56.1(13.23)-57.9(9.47)	22/8-23/7	4	MOCA, MBI
Zucchella C 2014[29]	Italy	CA-NOR	42-45	64(56,74)-70(62,76)	23/19-23/22	4	MMSE
Prokopenko SV 2013[30]	Russia	CA-NOR	24-19	61(57, 69)-66(61, 69)	13/11-10/9	2	MOCA, MMSE
Song HY 2020[31]	China	tDCS-NOR	15-15	64.76(8.65)	17/13	NG	MOCA, MBI
Tong JX 2019[32]	China	tDCS-NOR	31-31	64.7(7.3)-64.4(7.9)	18/13-20/11	4	MOCA, MMSE
Jin J 2019[33]	China	tDCS-NOR	45-45	53.1(5.3)-52.2(5.2)	24/21-23/22	8	MOCA, MBI
Mi Y 2019[34]	China	tDCS-NOR	15-15	52.80(7.27)-52.93(10.71)	8/7-9/6	3	MOCA, MBI
Yun GJ 2015[35]	Korea	tDCS-NOR	30-15	59.9(13.78)-68.5(14.6)	13/17-7/8	3	MMSE, MBI
Yu YL 2021[36]	China	Acupuncture-NOR	30-30	59(3)-59(3)	18/12-17/13	4	MOCA
Xiong J 2020[37]	China	Acupuncture-NOR	35-35	63.0(7.23)-65.3(8.52)	20/15-17/18	8	MMSE
Chen AZ 2020[38]	China	Acupuncture-NOR	30-30	62.00(5.12)-61.77(4.81)	13/17-14/16	4	MOCA, MBI
Yao R 2020[39]	China	Acupuncture-NOR	30-30	54.6(11.8)-57.4(12.8)	19/11-21/9	4	MOCA, MMSE
Feng L 2020[40]	China	Acupuncture-NOR	30-30	65.77(5.25)-64.3(5.98)	17/13-16/14	8	MMSE
Mao CX 2019[41]	China	Acupuncture-NOR	39-39	57.3(15.6)-58.2(16.2)	18/21-19/20	6	MOCA, MBI

Yang Y 2019[42]	China	Acupuncture-NOR	40-40	51.35(7.30)-51.72(7.46)	24/16-22/18	4	MMSE, MBI
Wang W 2018[43]	China	Acupuncture-NOR	50-50	53.8(11.7)-54.5(13.6)	24/26-26/24	3	MOCA, MMSE, MBI
Pu Y 2018[44]	China	Acupuncture-NOR	53-53	60.37(5.45)-59.15(5.29)	33/20-34/19	12	MMSE
Yang M 2017[45]	China	Acupuncture-NOR	47-44	58.72(5.18)	52/39	4	MMSE, MBI
Yang F 2017[46]	China	Acupuncture-NOR	30-30	66.9(7.6)-68.8(8.1)	17/13-16/14	4	MMSE
Ding X 2016[47]	China	Acupuncture-NOR	40-46	56.24(8.12)-57.87(9.01)	24/16-24/22	8	MMSE, MBI
Chen L 2016[48]	China	Acupuncture-NOR	125-125	62.52(10.60)-64.06(10.54)	74/51-74/51	3	MOCA, MMSE
Liu J 2013[49]	China	Acupuncture-NOR	25-25	53.40-(8.48)	34/16	4	MMSE
Song QM 2010[50]	China	Acupuncture-NOR	60-60	72.2(7)-70(7.6)	24/36-20/40	3	MMSE
Huang F 2008[51]	China	Acupuncture-NOR	40-40	59.22(10.6)-61.05(9.68)	21/19-20/20	4	MMSE
park I 2015[52]	Korea	TMS-CA	10-10	NG	4/6-5/5	4	MMSE
Zhang HL 2019[53]	China	VR	34	64.8(8.9)	17/17	4	MMSE, MBI
		Acupuncture	34	64.3(9.2)	18/16		
		NOR	34	64.0(9.6)	17/17		
Luo WH 2019[54]	China	tDCS-CA	32-32	56.15(6.85)-55.85(7.02)	18/14-17/15	6	MOCA, MMSE
Jiang C 2016[55]	China	Acupuncture	52	61.58(9.71)	25/27	12	MOCA, MMSE
		CA	51	62.37(7.89)	25/26		
		NOR	49	60.53(9.19)	24/25		

TMS: Transcranial Magnetic Stimulation; VR: Virtual Reality Exposure Therapy; CA: Computer-assisted cognitive rehabilitation; tDCS: Transcranial Direct Current Stimulation; Acu: Acupuncture; NOR: Normal rehabilitation, including conventional rehabilitation and routine cognition training.

Reference：

[1] Huang CY. rTMS combined with tDCS in the treatment of memory dysfunction after stroke[D]. Tianjin Institute of Physical Education, 2021.

[2] MA J, LI H, ZHANG J, et al. Effects of low frequency repetitive transcranial magnetic stimulation combined with cognitive training on the thyroid hormone levels and cognitive function in patients with cognitive impairment after cerebral stroke[J]. Hebei Medicine, 2021,43(16): 2436-2441.

[3] Wang X, Liu JL, Wang D. Resting state fMRI study on therapeutic effect of rTMS for cognitive dysfunction in patients with hemorrhagic stroke[J]. Chinese Rehabilitation, 2021,36(08): 451-455.

[4] ZHANG J, MA J, LI H, et al. Effects of repetitive transcranial magnetic stimulation on post-stroke cognitive impairment and lipid metabolism[J]. Chinese Rehabilitation, 2021,36(10): 584-588.

[5] LIU Y, YIN M, LUO J, et al. Effects of transcranial magnetic stimulation on the performance of the activities of daily living and attention function after stroke: a randomized controlled trial[J]. Clinical Rehabilitation, 2020,34(12): 1465-1473.

[6] Chen ZY, Gong JQ, Wu YF, et al. Effect of repeated transcranial magnetic stimulation combined with cognitive rehabilitation training on cognitive impairment after stroke[J]. Chinese journal of Physical Medicine and Rehabilitation, 2019(03): 199-201.

[7] Zheng J, Shi JJ, Gu Li Ping, et al. Therapeutic effects of high frequency repetitive transcranial magnetic stimulation in treating vascular cognitive impairment after stroke but no dementia[J]. Chinese Rehabilitation, 2017,32(06): 488-491.

[8] Hu LM. Effect of repeated transcranial magnetic stimulation combined with rehabilitation training on patients with vascular cognitive impairment without dementia[D]. Qingdao University, 2016.

[9] Xu QQ, Cao ZY, Zhang QH. Effect of repeated transcranial magnetic stimulation combined with cognitive training on vascular cognitive impairment without dementia[J]. Journal of Modern Integrated Chinese and Western Medicine, 2018,27(34): 3768-3771.

[10] FARIA A L, PINHO M S, BERMÚDEZ I BADIA S. A comparison of two personalization and adaptive cognitive rehabilitation approaches: a randomized controlled trial with chronic stroke patients[J]. Journal of NeuroEngineering and Rehabilitation, 2020,17(1): 78.

[11] Ren YG, Li JY, Hu K, et al. Therapeutic effect of virtual reality training on cognitive dysfunction after cerebral infarction[J]. International Journal of Psychiatry, 2020,47(06): 1193-1195.

[12] Guo JQ, Dong J. Effect of virtual reality training on cognitive impairment after cerebral apoplexy[J]. Women's Health Study of Chinese and foreign , 2020(24): 111-112.

[13] Xue H. Effects of neuroelectrophysiological monitoring virtual reality training on cognitive function and limb motor function in convalescent patients with stroke[J]. Modern practical medicine, 2020,32(06): 613-615.

[14] OH Y, KIM G, HAN K, et al. Efficacy of Virtual Reality Combined With Real Instrument Training for Patients With Stroke: A Randomized Controlled Trial[J]. Archives of Physical Medicine and Rehabilitation, 2019,100(8): 1400-1408.
[15] ROGERS J M, DUCKWORTH J, MIDDLETON S, et al. Elements virtual rehabilitation improves motor, cognitive, and functional outcomes in adult stroke: evidence from a randomized controlled pilot study[J]. Journal of NeuroEngineering and Rehabilitation, 2019,16(1): 56.
[16] Fu KJ, Sun LN, Fan F, et al. Effect of virtual reality training on cognitive function of convalescent patients with cerebral infarction[J]. Chinese journal of Physical Medicine and Rehabilitation, 2019(09): 682-684.
[17] Xiao X, Liang B. Effects of virtual reality training on cognitive function and P300 in convalescent stroke patients[J]. Chinese Journal of Rehabilitation Medicine, 2019,34(03): 339-341.
[18] FARIA A L, CAMEIRÃO M S, COURAS J F, et al. Combined Cognitive-Motor Rehabilitation in Virtual Reality Improves Motor Outcomes in Chronic Stroke – A Pilot Study[J]. Frontiers in Psychology, 2018,9: 854.
[19] Hu YQ, Li B, Wang JY, et al. Effects of short-term virtual reality rehabilitation training combined with cognitive intervention on motor function, Lovett muscle strength grade and quality of life in elderly patients with hemiplegia after stroke[J]. Chinese Journal of Medical Frontiers (electronic edition), 2018,10(08): 97-101.
[20] FARIA A L, ANDRADE A, SOARES L, et al. Benefits of virtual reality based cognitive rehabilitation through simulated activities of daily living: a randomized controlled trial with stroke patients[J]. Journal of NeuroEngineering and Rehabilitation, 2016,13(1): 96.
[21] Zhang L, Liu C, Li HM, et al. Effects of computer aided cognitive rehabilitation system on cognitive impairment in stroke patients[J]. Modern Medicine, 2020,48(10): 1332-1334.
[22] Luo XJ, Shi T, Liu Q. Effects of computerized cognitive training on cognitive dysfunction after stroke[J]. Stroke and Nervous Diseases, 2020,27(06): 821-823.
[23] Liu RF, Zhou J, Zeng Q, et al. Effects of computer aided cognitive training on cognitive impairment after stroke[J]. Guangdong Medical, 2019,40(10): 1401-1404.
[24] Chen JJ, Mu YF, Huang XP, et al. Effects of computer aided cognitive training on attention disorder in stroke patients[J]. Anhui Medical, 2019,40(08): 865-868.
[25] Lu CH, Wang K, Wu ZH, et al. Effects of digital OT cognitive function training on cognition, upper limb movement and daily living activities of stroke patients[J]. Modern medicine, 2019,47(04): 373-376.
[26] LIU X, HUANG X A, LIN J, et al. Computer Aided Technology-Based Cognitive Rehabilitation Efficacy Against Patients' Cerebral Stroke[J]. NeuroQuantology, 2018,16(4): 86-92.
[27] De LUCA R, LEONARDI S, SPADARO L, et al. Improving Cognitive Function in Patients with Stroke: Can Computerized Training Be the Future?[J]. Journal of Stroke and Cerebrovascular Diseases, 2018,27(4): 1055-1060.

[28] Xiao ST. Effects of computer-aided cognitive training based on information processing theory on cognitive impairment after stroke[D]. Fujian University of Traditional Chinese Medicine, 2018.
[29] ZUCCHELLA C, CAPONE A, CODELLA V, et al. Assessing and restoring cognitive functions early after stroke[J]. Funct Neurol, 2014,29(4): 255-262.
[30] PROKOPENKO S V, MOZHEYKO E Y, PETROVA M M, et al. Correction of post-stroke cognitive impairments using computer programs[J]. Journal of the Neurological Sciences, 2013,325(1-2): 148-153.
[31] Song HY. Effects of transcranial direct current stimulation on cognitive function in stroke patients[J]. Chinese Journal of Modern Medicine, 2020,30(03): 113-116.
[32] Tong JX, Chen Y, Liu H, et al. Effects of transcranial direct current stimulation combined with cognitive function training on cognition and daily living activities of patients with cerebral infarction[J]. Massage and Rehabilitation Medicine, 2019,10(12): 17-19.
[33] Jin J, Jiang S, Pan XL, et al. Effects of transcranial direct current stimulation combined with rehabilitation training on cognitive function and limb motor function of stroke patients with hemiplegia[J]. Chinese journal of Physical Medicine and Rehabilitation, 2019(06): 415-417.
[34] Mi Y. Clinical study of transcranial direct current stimulation on executive dysfunction after stroke[D]. Shanxi Medical University, 2019.
[35] YUN G J, CHUN M H, KIM B R. The Effects of Transcranial Direct-Current Stimulation on Cognition in Stroke Patients[J]. Journal of Stroke, 2015,17(3): 354-358.
[36] Yu YL, Cheng MD, Ma M, et al. Effect of Bo's abdominal acupuncture on brain RESTING state F MRI in patients with cognitive dysfunction after stroke[J]. Shanghai Journal of Acupuncture, 2021,40(11): 1293-1298.
[37] XIONG J, ZHANG Z, MA Y, et al. The effect of combined scalp acupuncture and cognitive training in patients with stroke on cognitive and motor functions[J]. NeuroRehabilitation, 2020,46(1): 75-82.
[38] Chen A Z, Lin ZC, Lan F. Effects of head acupuncture combined with cognitive rehabilitation training platform on cognitive function after stroke[J]. Massage and Rehabilitation Medicine, 2020,11(05): 22-23.
[39] Yao R, Gong ZK, Zhang KW. Clinical study of Jin SAN Acupuncture therapy on cognitive impairment after stroke[J]. Acupuncture and Massage Medicine, 2020,18(1): 40-46.
[40] Feng L. Clinical study of zhi Qi acupuncture therapy on mild vascular dementia after cerebral apoplexy[D]. Zhejiang Chinese Medicine University, 2020.
[41] Mao CX, Cheng FN. Cephalic acupuncture combined with modern rehabilitation training for 39 cases of post-stroke cognitive impairment[J]. Traditional Chinese Medicinal Research, 2019,32(10): 47-49.
[42] Yang Y, Zhang H. Clinical effect of tonifying kidney and enriching marrow acupuncture combined with rehabilitation training on cognitive dysfunction after stroke and its influence on daily living activities[J]. Clinical Journal of Acupuncture, 2019,35(07): 24-27.

[43] Wang W, Lu XY, Zhu LS, et al. Effects of scalp acupuncture combined with rehabilitation training on cognitive dysfunction in stroke patients[J]. New traditional Chinese medicine, 2018,50(03): 164-167.
[44] Pu Y. Effect of acupuncture on cognitive dysfunction after cerebral infarction[J]. Shenzhen Journal of Integrated Traditional Chinese and Western Medicine, 2018,28(24): 34-36.
[45] Yang M, Zhang P. Therapeutic effect of acupuncture combined with cognitive function training on 47 cases of cognitive impairment after stroke[J]. Yunnan Journal of Traditional Chinese Medicine, 2017,38(02): 64-66.
[46] YANG F, LUO K T, ZHU J H, et al. Effect of long-time needle retaining at Baihui (GV 20) on cognitive impairment in post-stroke patients[J]. JOURNAL OF ACUPUNCTURE AND TUINA SCIENCE, 2017,15(6): 398-402.
[47] Ding X, Zhang H. Effects of cephalic acupuncture combined with motor relearning on cognitive impairment in patients after stroke[J]. Hebei Traditional Chinese Medicine, 2016,38(04): 586-588.
[48] CHEN L, FANG J, MA R, et al. Additional effects of acupuncture on early comprehensive rehabilitation in patients with mild to moderate acute ischemic stroke: a multicenter randomized controlled trial[J]. BMC Complement Altern Med, 2016,16: 226.
[49] Liu J, Feng XD. Clinical study of electroacupuncture baihui and Shenting point combined with rehabilitation training on cognitive impairment after stroke[J]. Journal of traditional Chinese medicine, 2013,28(04): 608-610.
[50] Song QM. Clinical observation on acupuncture treatment of vascular cognitive impairment in stroke[D]. Guangzhou University of Chinese Medicine, 2010.
[51] Huang F, Liu Y, Zhou FX, et al. Effect of acupuncture on vascular cognitive impairment after cerebral infarction[J]. Guangdong Medical, 2008(11): 1918-1920.
[52] PARK I, YOON J. The effect of computer-assisted cognitive rehabilitation and repetitive transcranial magnetic stimulation on cognitive function for stroke patients[J]. Journal of Physical Therapy Science, 2015,27(3): 773-776.
[53] Zhang HL, Che WS, Chu NN. Effect of head acupuncture combined with virtual scene interactive training on functional rehabilitation of stroke patients[J]. Chinese Journal of Gerontology, 2019,39(20): 4902-4906.
[54] Luo WH, Huang XH, Ling SQ. Effect of transcranial direct current stimulation combined with computer-assisted cognitive rehabilitation training on cognitive dysfunction after stroke[J]. Journal of China Prescription Drug, 2019,17(02): 119-121.
[55] JIANG C, YANG S, TAO J, et al. Clinical Efficacy of Acupuncture Treatment in Combination With RehaCom Cognitive Training for Improving Cognitive Function in Stroke: A 2 × 2 Factorial Design Randomized Controlled Trial[J]. Journal of the American Medical Directors Association, 2016,17(12): 1114-1122.

Supplementary Table 3. The evaluating of model's convergence

**MOCA**	
**Parameter**	**PSRF**
d.NOR.ACU	1.00
d.NOR.CA	1.00
d.NOR.TMS	1.00
d.NOR.VR	1.00
d.NOR.tDCS	1.00
sd.d	1.00
**MMSE**	
**Parameter**	**PSRF**
d.NOR.ACU	1.00
d.NOR.CA	1.00
d.NOR.TMS	1.00
d.NOR.VR	1.00
d.NOR.tDCS	1.00
sd.d	1.00
**BI**	
**Parameter**	**PSRF**
d.NOR.ACU	1.00
d.NOR.CA	1.00
d.NOR.TMS	1.00
d.NOR.VR	1.00
d.NOR.tDCS	1.00
sd.d	1.00

PSRF: The potential scale reduction factor. TMS: Transcranial Magnetic Stimulation; VR: Virtual Reality Exposure Therapy; CA: Computer-assisted cognitive rehabilitation; tDCS: Transcranial Direct Current Stimulation; Acu: Acupuncture; NOR: Normal rehabilitation, including conventional rehabilitation and routine cognition training.

Supplementary Table 4. The SUCRA value

**Treatment**	**SUCRA** (MOCA)	**SUCRA** (MMSE)	**SUCRA** (BI)
NOR	1.5	9.5	4.2
TMS	79.7	76.1	89.1
VR	25.4	59.4	50.6
CA	44.4	42.7	53.7
tDCS	64.4	40.3	74.2
Acupuncture	84.7	72.1	28.1

SUCRA: The surface under the cumulative ranking area. TMS: Transcranial Magnetic Stimulation; VR: Virtual Reality Exposure Therapy; CA: Computer-assisted cognitive rehabilitation; tDCS: Transcranial Direct Current Stimulation; Acu: Acupuncture; NOR: Normal rehabilitation, including conventional rehabilitation and routine cognition training.

Supplementary Table 5. Summary of Adverse Effects

Study	Intervention	Adverse Effects
Cheng ZY 2019	TMS-NOR	no adverse effects
Zheng J 2017	TMS-NOR	2 cases of mild headache and dizziness
Hu LM 2016	TMS-NOR	2 cases of mild headache and dizziness (resolved without treatment)
Rogers JM 2019	VR-NOR	no adverse effects
Jin J 2019	tDCS-NOR	Itching, tingling and burning at the site of electrical stimulation for a short time in part of the patients
Feng L 2020	ACU-NOR	3 cases of scalp haematoma after acupuncture
Xiao ST 2018	CA-NOR	no adverse effects

TMS: Transcranial Magnetic Stimulation; VR: Virtual Reality Exposure Therapy; CA: Computer-assisted cognitive rehabilitation; tDCS: Transcranial Direct Current Stimulation; Acu: Acupuncture; NOR: Normal rehabilitation, including conventional rehabilitation and routine cognition training.

Supplementary Table 6. Assessment of inconsistency (Node-splitting model)

Nodes	Direct		Indirect		Difference		P value
	logor	SE	logor	SE	logor	SE	
**MoCA**							
NOR-CA	2.18	0.68	3.04	1.94	-0.869	2.06	0.674
NOR-tDCS	2.88	0.92	3.12	2.13	-0.237	2.32	0.919
NOR-Acu	3.68	0.75	3.20	3.84	0.47	3.91	0.902
CA-tDCS	0.83	2.02	0.59	1.14	0.23	2.32	0.918
CA-Acu	0.14	2.10	1.69	1.05	1.55	2.35	0.510
**MMSE**							
NOR-TMS	2.93	1.28	0.20	3.17	2.73	3.42	0.425
NOR-VR	1.72	1.05	4.61	5.25	-2.89	5.35	0.589
NOR-CA	0.72	1.31	2.64	2.18	-1.91	2.54	0.451
NOR-tDCS	1.03	2.12	1.07	3.19	-0.03	3.83	0.992
NOR-Acu	2.31	0.74	1.75	3.94	0.56	4.01	0.889
TMS-CA	0 .70	2.93	-2.03	1.76	2.73	3.42	0.425
VR-Acu	-0.40	2.91	0.65	1.36	1.05	3.21	0.742
CA-tDCS	-0.16	2.95	-0.21	2.44	0.04	3.83	0.991
CA-Acu	0.22	2.92	1.26	1.46	-1.04	3.27	0.750
BI							
NOR-VR	5.88	1.9	21.21	7.11	-15.33	7.36	**0.037**
NOR-Acu	9.85	1.70	5.58	8.96	4.27	9.12	0.640
VR-Acu	-1.93	3.91	5.37	2.92	-7.3	4.88	0.135

TMS: Transcranial Magnetic Stimulation; VR: Virtual Reality Exposure Therapy; CA: Computer-assisted cognitive rehabilitation; tDCS: Transcranial Direct Current Stimulation; Acu: Acupuncture; NOR: Normal rehabilitation, including conventional rehabilitation and routine cognition training.

Supplementary Table 7. Evaluation of the quality of evidence using GRADE framework

Comparison	Study Limitations	Inconsistency	Indirectness	Imprecision	Publication Bias	GRADE
**MOCA**						
TMS-NOR	serious	serious	not serious	not serious	not serious	LOW
VR-NOR	serious	not serious	not serious	serious	not serious	LOW
CA-NOR	serious	serious	not serious	not serious	not serious	LOW
tDCS-NOR	serious	serious	not serious	not serious	not serious	LOW
ACU-NOR	serious	serious	not serious	not serious	not serious	LOW
CA-tDCS	serious	not serious	not serious	serious	not serious	LOW
CA-ACU	serious	not serious	not serious	not serious	not serious	Moderate
TMS-VR	serious	not serious	not serious	serious	not serious	LOW
TMS-CA	serious	not serious	not serious	serious	not serious	LOW
TMS-tDCS	serious	not serious	not serious	serious	not serious	LOW
TMS-ACU	serious	not serious	not serious	not serious	not serious	Moderate
VR-CA	serious	not serious	not serious	serious	not serious	LOW
VR-tDCS	serious	not serious	not serious	serious	not serious	LOW
VR-ACU	serious	not serious	not serious	serious	not serious	LOW
tDCS-ACU	serious	not serious	not serious	serious	not serious	LOW
**MMSE**						
TMS-NOR	serious	serious	not serious	not serious	not serious	LOW

VR-NOR	very serious	not serious	not serious	serious	not serious	VERY LOW
CA-NOR	serious	not serious	not serious	serious	not serious	LOW
tDCS-NOR	serious	not serious	not serious	serious	not serious	LOW
ACU-NOR	serious	serious	not serious	not serious	not serious	LOW
TMS-CA	serious	not serious	not serious	serious	not serious	LOW
VR-ACU	serious	not serious	not serious	serious	not serious	LOW
CA-tDCS	serious	not serious	not serious	serious	not serious	LOW
CA-ACU	serious	not serious	not serious	serious	not serious	LOW
TMS-VR	serious	not serious	not serious	serious	not serious	LOW
TMS-tDCS	serious	not serious	not serious	serious	not serious	LOW
TMS-ACU	serious	not serious	not serious	serious	not serious	LOW
VR-CA	serious	not serious	not serious	serious	not serious	LOW
VR-tDCS	serious	not serious	not serious	serious	not serious	LOW
tDCS-ACU	serious	not serious	not serious	serious	not serious	LOW
**BI**						
TMS-NOR	serious	serious	not serious	not serious	not serious	LOW
VR-NOR	very serious	not serious	not serious	not serious	not serious	LOW
CA-NOR	very serious	serious	not serious	not serious	not serious	VERY LOW
tDCS-NOR	serious	serious	not serious	not serious	not serious	LOW

ACU-NOR	serious	serious	not serious	not serious	not serious	LOW
VR-ACU	serious	not serious	not serious	serious	not serious	LOW
TMS-VR	serious	not serious	not serious	serious	not serious	LOW
TMS-CA	serious	not serious	not serious	serious	not serious	LOW
TMS-tDCS	serious	not serious	not serious	serious	not serious	LOW
TMS-ACU	serious	not serious	not serious	serious	not serious	LOW
VR-CA	very serious	not serious	not serious	serious	not serious	VERY LOW
VR-tDCS	very serious	not serious	not serious	serious	not serious	VERY LOW
CA-tDCS	serious	not serious	not serious	serious	not serious	LOW
CA-ACU	very serious	not serious	not serious	serious	not serious	VERY LOW
tDCS-ACU	serious	not serious	not serious	serious	not serious	LOW

TMS: Transcranial Magnetic Stimulation; VR: Virtual Reality Exposure Therapy; CA: Computer-assisted cognitive rehabilitation; tDCS: Transcranial Direct Current Stimulation; Acu: Acupuncture; NOR: Normal rehabilitation, including conventional rehabilitation and routine cognition training.

2. Supplementary Figures

Study or Subgroup	TMS Mean	TMS SD	TMS Total	NOR Mean	NOR SD	NOR Total	Weight	Mean Difference IV, Random, 95% CI
Cheng ZY 2019	6.75	2.95	70	2.44	2.86	70	13.8%	4.31 [3.35, 5.27]
Huang CY 2021	5.33	3.8	12	2.75	4.48	12	8.8%	2.58 [-0.74, 5.90]
Hu LM 2016	4.77	1.55	30	2.6	1.54	30	14.0%	2.17 [1.39, 2.95]
Ma J 2021	4.73	3.83	37	3.13	4.18	38	12.2%	1.60 [-0.21, 3.41]
Wang X 2021	7.92	3.3	60	3.68	2.96	60	13.5%	4.24 [3.12, 5.36]
Xu QQ 2018	4.04	3.16	33	1.69	3.04	35	12.9%	2.35 [0.87, 3.83]
Zhang J 2021	5.31	4.01	21	3.31	3.78	22	11.0%	2.00 [-0.33, 4.33]
Zheng J 2017	9.27	1.43	30	2	2.15	30	13.8%	7.27 [6.35, 8.19]
**Total (95% CI)**			**293**			**297**	**100.0%**	**3.42 [1.86, 4.98]**

Heterogeneity: Tau² = 4.35; Chi² = 84.53, df = 7 (P < 0.00001); I² = 92%
Test for overall effect: Z = 4.30 (P < 0.0001)

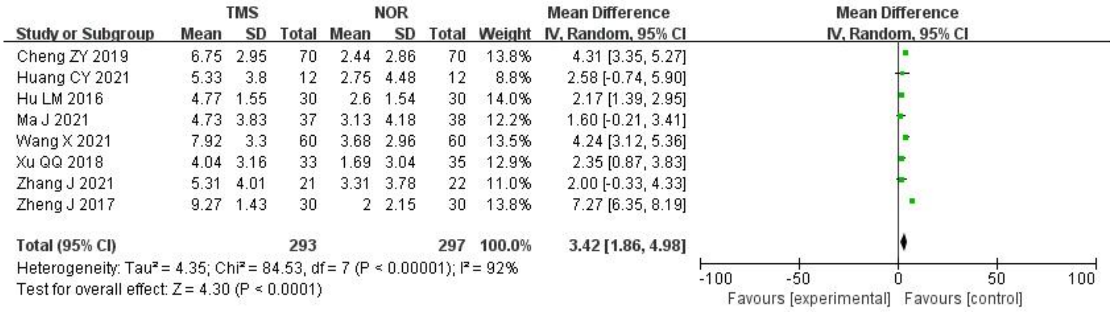

Study or Subgroup	tDCS Mean	tDCS SD	tDCS Total	NOR Mean	NOR SD	NOR Total	Weight	Mean Difference IV, Random, 95% CI
Jin J 2019	9.9	2.52	45	4.8	1.67	45	26.1%	5.10 [4.22, 5.98]
Mi Y 2019	5.87	2.28	15	3.73	1.86	15	23.2%	2.14 [0.65, 3.63]
Song HY 2020	2.12	0.42	15	0.19	0.5	15	27.7%	1.93 [1.60, 2.26]
Tong JX 2019	5	3.4	31	2.7	2.65	31	23.0%	2.30 [0.78, 3.82]
**Total (95% CI)**			**106**			**106**	**100.0%**	**2.89 [1.15, 4.63]**

Heterogeneity: Tau² = 2.82; Chi² = 43.46, df = 3 (P < 0.00001); I² = 93%
Test for overall effect: Z = 3.26 (P = 0.001)

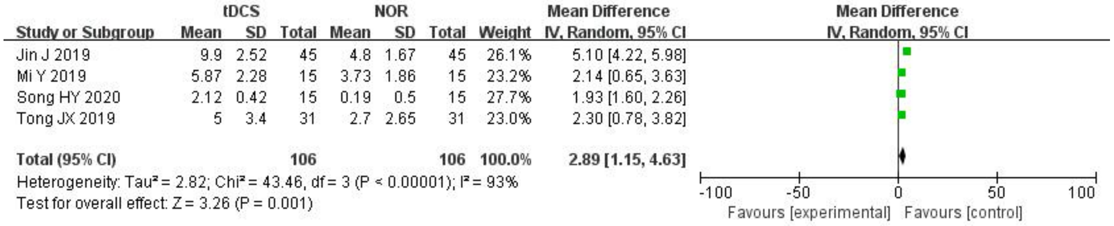

Study or Subgroup	VR Mean	VR SD	VR Total	NOR Mean	NOR SD	NOR Total	Weight	Mean Difference IV, Fixed, 95% CI
Faria AL 2018	2.5	5.29	12	2.5	5	12	4.4%	0.00 [-4.12, 4.12]
Faria AL 2020	2	4.07	14	0	4.49	18	8.4%	2.00 [-0.97, 4.97]
Oh Y 2019	1.8	4.2	17	1.7	3.8	14	9.3%	0.10 [-2.72, 2.92]
Ren YG 2020	4.93	2.63	40	4.39	2.26	40	64.1%	0.54 [-0.53, 1.61]
Rogers JM 2019	6.4	2.55	10	2.2	3.87	10	9.0%	4.20 [1.33, 7.07]
Xiao X 2019	5.01	4.99	16	3.96	6.63	18	4.8%	1.05 [-2.87, 4.97]
**Total (95% CI)**			**109**			**112**	**100.0%**	**0.95 [0.09, 1.81]**

Heterogeneity: Chi² = 6.51, df = 5 (P = 0.26); I² = 23%
Test for overall effect: Z = 2.17 (P = 0.03)

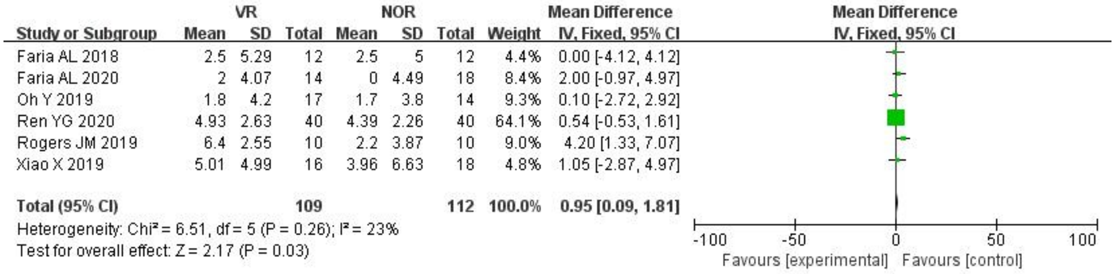

Study or Subgroup	CA Mean	CA SD	CA Total	NOR Mean	NOR SD	NOR Total	Weight	Mean Difference IV, Random, 95% CI
Chen JJ 2019	5.58	2.67	24	2.12	3.41	24	12.4%	3.46 [1.73, 5.19]
Jiang C 2016	2.77	5.19	51	1.32	5.81	49	11.2%	1.45 [-0.71, 3.61]
Liu RF 2019	6	2.2	15	1.93	2.03	15	12.9%	4.07 [2.56, 5.58]
Liu X 2018	10.04	3.45	62	5	3.11	66	13.8%	5.04 [3.90, 6.18]
Lu CH 2019	1.56	3	50	0.26	3.28	50	13.6%	1.30 [0.07, 2.53]
Luo XJ 2020	5.23	3.2	30	3.86	2.87	30	12.9%	1.37 [-0.17, 2.91]
Prokopenko SV 2013	4	2.56	24	4.5	2.89	19	12.6%	-0.50 [-2.15, 1.15]
Xiao ST 2018	3.8	4.16	30	3.16	5.05	30	10.8%	0.64 [-1.70, 2.98]
**Total (95% CI)**			**286**			**283**	**100.0%**	**2.17 [0.74, 3.60]**

Heterogeneity: Tau² = 3.54; Chi² = 46.04, df = 7 (P < 0.00001); I² = 85%
Test for overall effect: Z = 2.97 (P = 0.003)

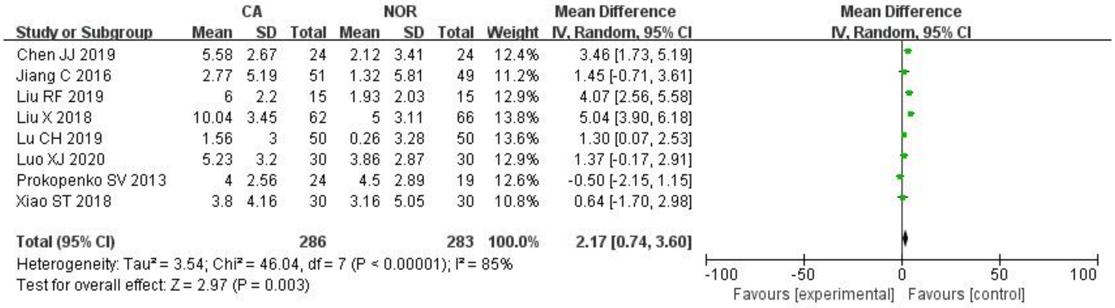

Study or Subgroup	Acupuncture Mean	Acupuncture SD	Acupuncture Total	NOR Mean	NOR SD	NOR Total	Weight	Mean Difference IV, Random, 95% CI
Chen AZ 2020	7.72	2.05	30	3.17	1.84	30	15.6%	4.55 [3.56, 5.54]
Chen L 2016	1.75	2.19	125	1.21	2.25	125	16.0%	0.54 [-0.01, 1.09]
Jiang C 2016	2.83	6.84	52	1.32	5.81	49	13.4%	1.51 [-0.96, 3.98]
Mao CX 2019	10.3	3.36	39	3.62	2.99	39	15.1%	6.68 [5.27, 8.09]
Wang W 2018	11.64	4.69	50	7.08	3.57	50	14.8%	4.56 [2.93, 6.19]
Yao R 2020	7.77	6.57	30	3.6	6.02	30	12.0%	4.17 [0.98, 7.36]
Yu YL 2021	7.65	5.51	30	3.73	4.95	30	13.0%	3.92 [1.27, 6.57]
**Total (95% CI)**			**356**			**353**	**100.0%**	**3.70 [1.51, 5.89]**

Heterogeneity: Tau² = 7.71; Chi² = 106.76, df = 6 (P < 0.00001); I² = 94%
Test for overall effect: Z = 3.31 (P = 0.0009)

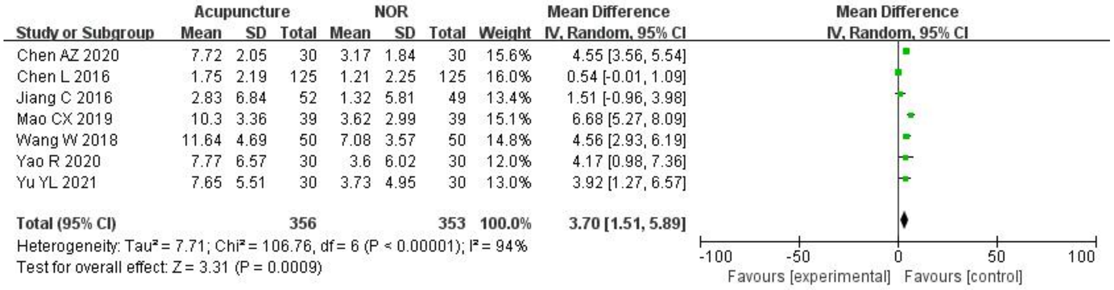

Study or Subgroup	Acupuncture Mean	Acupuncture SD	Acupuncture Total	CA Mean	CA SD	CA Total	Weight	Mean Difference IV, Random, 95% CI
Jiang C 2016	2.83	6.84	52	2.77	5.19	51	100.0%	0.06 [-2.28, 2.40]
**Total (95% CI)**			**52**			**51**	**100.0%**	**0.06 [-2.28, 2.40]**

Heterogeneity: Not applicable
Test for overall effect: Z = 0.05 (P = 0.96)

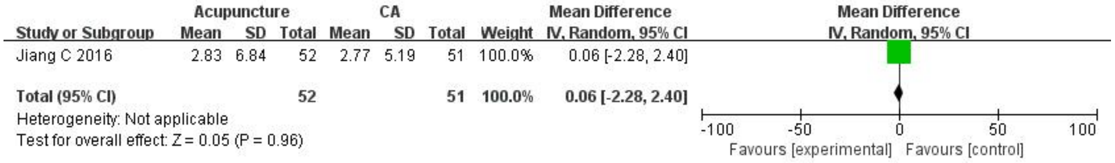

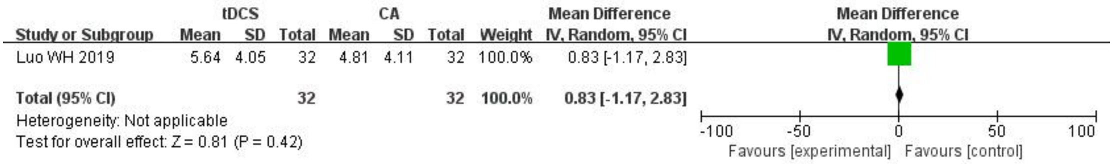

Supplementary Figure 1. Forest plots of the pairwise meta-analysis for MoCA. TMS: Transcranial Magnetic Stimulation; VR: Virtual Reality Exposure Therapy; CA: Computer-assisted cognitive rehabilitation; tDCS: Transcranial Direct Current Stimulation; Acu: Acupuncture; NOR: Normal rehabilitation, including conventional rehabilitation and routine cognition training.

Study or Subgroup	TMS Mean	TMS SD	TMS Total	NOR Mean	NOR SD	NOR Total	Weight	Mean Difference IV, Random, 95% CI
Hu LM 2016	1.57	1.43	30	1.34	1.28	30	22.0%	0.23 [-0.46, 0.92]
Liu Y 2020	3.07	4.18	29	1.2	3.37	29	18.8%	1.87 [-0.08, 3.82]
Wang X 2021	6.46	4.14	60	4.26	3.78	60	20.4%	2.20 [0.78, 3.62]
Zhang J 2021	6.38	4.07	21	3.82	4.36	22	17.0%	2.56 [0.04, 5.08]
Zheng J 2017	7.57	1.91	30	3.07	1.27	30	21.8%	4.50 [3.68, 5.32]
**Total (95% CI)**			**170**			**171**	**100.0%**	**2.27 [0.18, 4.36]**

Heterogeneity: Tau² = 5.04; Chi² = 61.43, df = 4 (P < 0.00001); I² = 93%
Test for overall effect: Z = 2.13 (P = 0.03)

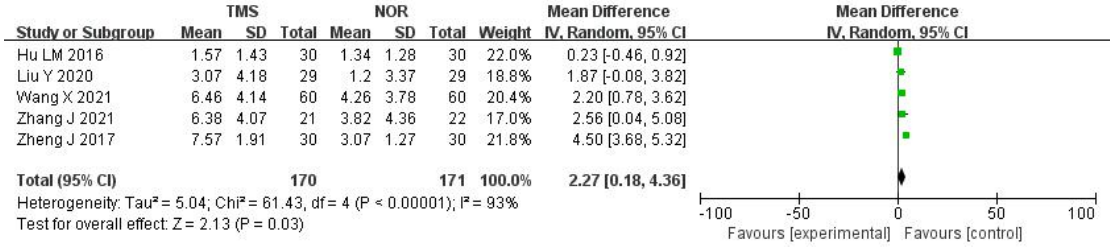

Study or Subgroup	tDCS Mean	tDCS SD	tDCS Total	NOR Mean	NOR SD	NOR Total	Weight	Mean Difference IV, Fixed, 95% CI
Faria AL 2018	3.05	4.47	30	2.7	4.75	15	18.4%	0.35 [-2.54, 3.24]
Xiao X 2019	4.9	2.55	31	3.3	2.95	31	81.6%	1.60 [0.23, 2.97]
**Total (95% CI)**			**61**			**46**	**100.0%**	**1.37 [0.13, 2.61]**

Heterogeneity: Chi² = 0.59, df = 1 (P = 0.44); I² = 0%
Test for overall effect: Z = 2.17 (P = 0.03)

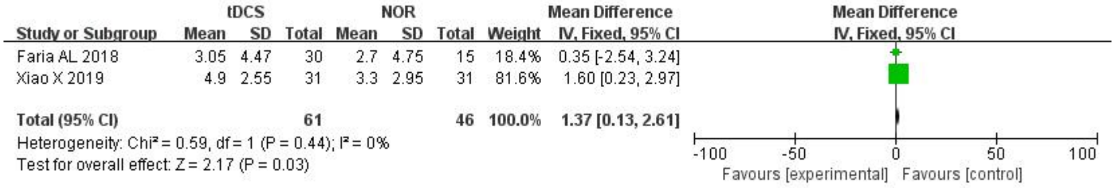

Study or Subgroup	VR Mean	VR SD	VR Total	NOR Mean	NOR SD	NOR Total	Weight	Mean Difference IV, Random, 95% CI
Faria AL 2016	6	3.64	9	3	4.07	9	6.9%	3.00 [-0.57, 6.57]
Fu KJ 2019	9.83	2.85	18	8.55	3.7	18	11.5%	1.28 [-0.88, 3.44]
Guo JQ 2020	5.61	2.45	25	2.93	2.31	25	15.0%	2.68 [1.36, 4.00]
Hu YQ 2018	1.8	1.95	33	1.4	2.23	33	16.3%	0.40 [-0.61, 1.41]
Oh Y 2019	0.6	3.1	17	1	1.55	14	13.5%	-0.40 [-2.08, 1.28]
Xiao X 2019	4.43	4.93	16	2.68	4.99	18	7.5%	1.75 [-1.59, 5.09]
Xue H 2020	9.85	3.75	50	5.55	3.76	50	14.4%	4.30 [2.83, 5.77]
Zhang HL 2019	2.28	3.31	34	1.23	2.38	34	14.8%	1.05 [-0.32, 2.42]
**Total (95% CI)**			**202**			**201**	**100.0%**	**1.68 [0.49, 2.87]**

Heterogeneity: Tau² = 2.00; Chi² = 27.57, df = 7 (P = 0.0003); I² = 75%
Test for overall effect: Z = 2.76 (P = 0.006)

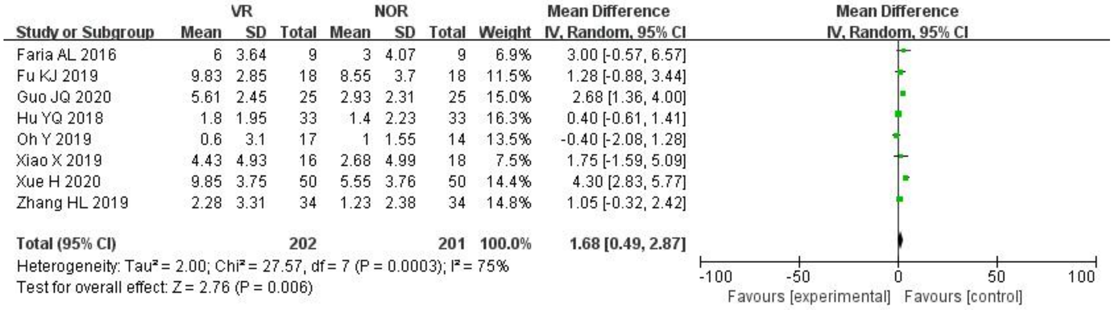

Study or Subgroup	CA Mean	CA SD	CA Total	NOR Mean	NOR SD	NOR Total	Weight	Mean Difference IV, Random, 95% CI
De Luca 2018	4.3	2.5	20	2.1	3.28	15	19.9%	2.20 [0.21, 4.19]
Jiang C 2016	2.84	3.96	51	1.59	4.19	49	20.8%	1.25 [-0.35, 2.85]
Prokopenko SV 2013	3	1.48	24	6.5	2.89	19	21.1%	-3.50 [-4.93, -2.07]
Zhang L 2020	1.68	5.08	37	-1.03	4.73	37	19.3%	2.71 [0.47, 4.95]
Zucchella C 2014	2.6	5.12	42	1.3	6.18	45	18.9%	1.30 [-1.08, 3.68]
**Total (95% CI)**			**174**			**165**	**100.0%**	**0.73 [-1.81, 3.26]**

Heterogeneity: Tau² = 7.37; Chi² = 36.89, df = 4 (P < 0.00001); I² = 89%
Test for overall effect: Z = 0.56 (P = 0.57)

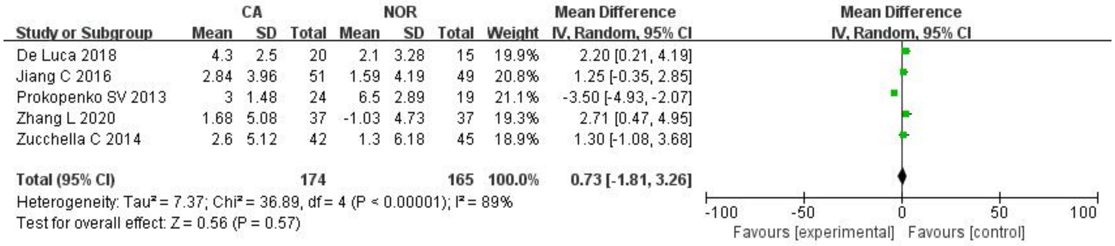

Study or Subgroup	Acupuncture Mean	Acupuncture SD	Acupuncture Total	NOR Mean	NOR SD	NOR Total	Weight	Mean Difference IV, Random, 95% CI
Chen L 2016	1.65	2.66	125	1.11	2.7	125	7.0%	0.54 [-0.12, 1.20]
Ding X 2016	6.88	3.22	40	2.61	4.15	46	6.7%	4.27 [2.71, 5.83]
Feng L 2020	4.97	2.34	30	2.23	1.51	30	6.9%	2.74 [1.74, 3.74]
Huang F 2008	4.33	3.89	40	11.54	4.24	40	6.5%	-7.21 [-8.99, -5.43]
Jiang C 2016	3.06	4.91	52	1.59	4.19	49	6.5%	1.47 [-0.31, 3.25]
Liu J 2013	15.08	1.14	25	8.8	0.96	25	7.0%	6.28 [5.70, 6.86]
Pu Y 2018	4.28	2.35	53	0.59	2.44	53	6.9%	3.69 [2.78, 4.60]
Song QM 2010	3	4.65	60	3	4.95	60	6.6%	0.00 [-1.72, 1.72]
Wang W 2018	12.68	4.98	50	3.44	1.87	50	6.7%	9.24 [7.77, 10.71]
Xiong J 2020	5.53	4.2	35	4.15	3.73	35	6.5%	1.38 [-0.48, 3.24]
Yang F 2017	7.27	2.54	30	3.8	2.21	30	6.8%	3.47 [2.27, 4.67]
Yang M 2017	4.12	4.27	47	1.92	4.33	44	6.6%	2.20 [0.43, 3.97]
Yang Y 2019	3.56	3.73	40	1.35	3.42	40	6.7%	2.21 [0.64, 3.78]
Yao R 2020	8.04	5.77	30	4.7	5.33	30	5.9%	3.34 [0.53, 6.15]
Zhang HL 2019	1.87	3.63	34	1.23	2.38	34	6.7%	0.64 [-0.82, 2.10]
**Total (95% CI)**			**691**			**691**	**100.0%**	**2.31 [0.65, 3.97]**

Heterogeneity: Tau² = 10.13; Chi² = 402.66, df = 14 (P < 0.00001); I² = 97%
Test for overall effect: Z = 2.73 (P = 0.006)

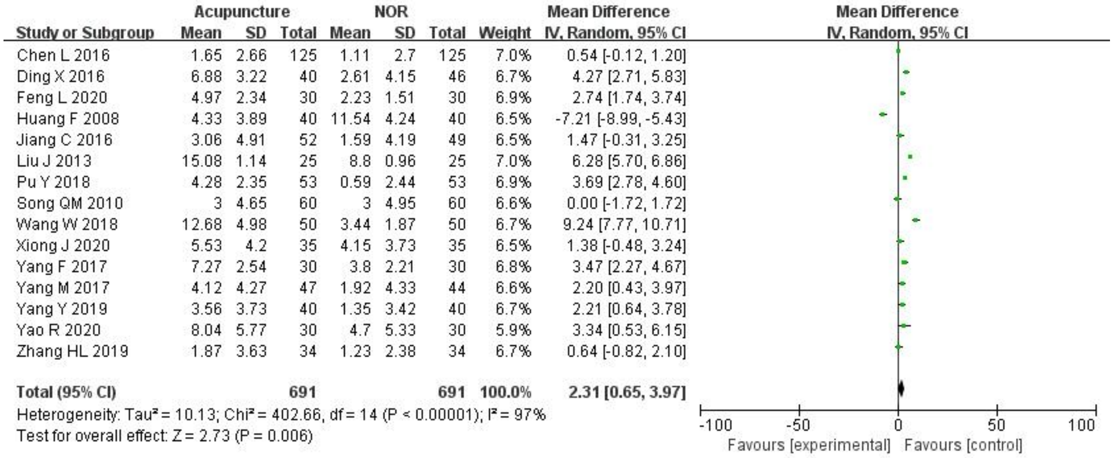

Study or Subgroup	Acupuncture Mean	Acupuncture SD	Acupuncture Total	CA Mean	CA SD	CA Total	Weight	Mean Difference IV, Random, 95% CI
Jiang C 2016	3.06	4.91	52	2.84	3.96	51	100.0%	0.22 [-1.50, 1.94]
**Total (95% CI)**			**52**			**51**	**100.0%**	**0.22 [-1.50, 1.94]**

Heterogeneity: Not applicable
Test for overall effect: Z = 0.25 (P = 0.80)

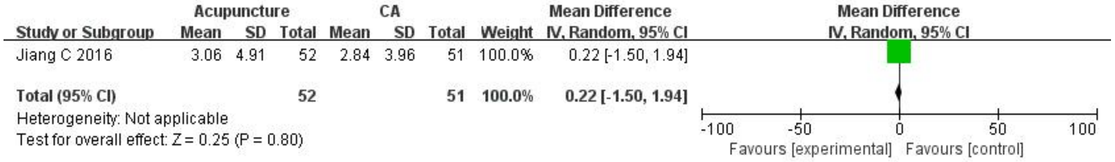

Study or Subgroup	tDCS Mean	tDCS SD	tDCS Total	CA Mean	CA SD	CA Total	Weight	Mean Difference IV, Random, 95% CI
Luo WH 2019	3.84	3.82	32	4.01	3.95	32	100.0%	-0.17 [-2.07, 1.73]
**Total (95% CI)**			**32**			**32**	**100.0%**	**-0.17 [-2.07, 1.73]**
Heterogeneity: Not applicable								
Test for overall effect: Z = 0.18 (P = 0.86)								

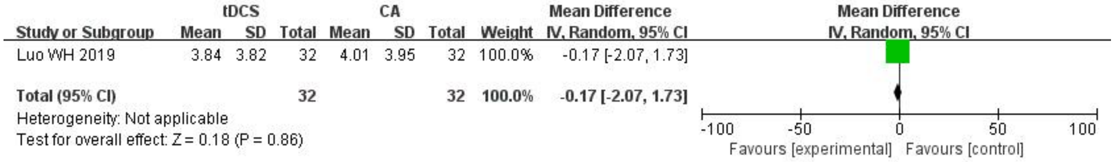

Study or Subgroup	TMS Mean	TMS SD	TMS Total	CA Mean	CA SD	CA Total	Weight	Mean Difference IV, Random, 95% CI
park I 2015	1.6	2.42	10	2.3	1.98	10	100.0%	-0.70 [-2.64, 1.24]
**Total (95% CI)**			**10**			**10**	**100.0%**	**-0.70 [-2.64, 1.24]**
Heterogeneity: Not applicable								
Test for overall effect: Z = 0.71 (P = 0.48)								

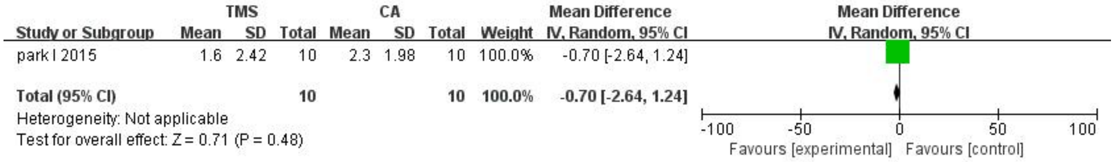

Study or Subgroup	VR Mean	VR SD	VR Total	Acupuncture Mean	Acupuncture SD	Acupuncture Total	Weight	Mean Difference IV, Random, 95% CI
Zhang HL 2019	2.28	3.31	34	1.87	3.63	34	100.0%	0.41 [-1.24, 2.06]
**Total (95% CI)**			**34**			**34**	**100.0%**	**0.41 [-1.24, 2.06]**
Heterogeneity: Not applicable								
Test for overall effect: Z = 0.49 (P = 0.63)								

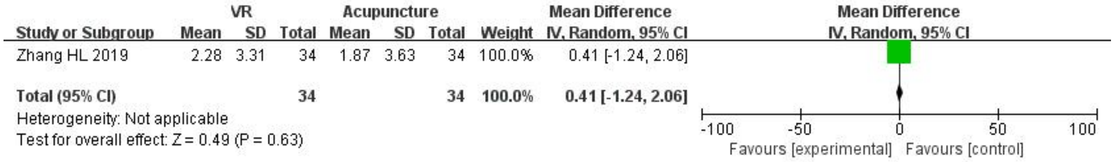

Supplementary Figure 2. Forest plots of the pairwise meta-analysis for MMSE. TMS: Transcranial Magnetic Stimulation; VR: Virtual Reality Exposure Therapy; CA: Computer-assisted cognitive rehabilitation; tDCS: Transcranial Direct Current Stimulation; Acu: Acupuncture; NOR: Normal rehabilitation, including conventional rehabilitation and routine cognition training.

Study or Subgroup	TMS Mean	TMS SD	TMS Total	NOR Mean	NOR SD	NOR Total	Weight	Mean Difference IV, Random, 95% CI
Cheng ZY 2019	18.3	12.76	70	7.6	11.78	70	32.9%	10.70 [6.63, 14.77]
Hu LM 2016	9	4.18	30	4.73	3.77	30	34.8%	4.27 [2.26, 6.28]
Zheng J 2017	25.83	9.81	30	6.6	8.24	30	32.3%	19.23 [14.65, 23.81]
**Total (95% CI)**			**130**			**130**	**100.0%**	**11.22 [2.53, 19.90]**

Heterogeneity: Tau² = 55.35; Chi² = 37.38, df = 2 (P < 0.00001); I² = 95%
Test for overall effect: Z = 2.53 (P = 0.01)

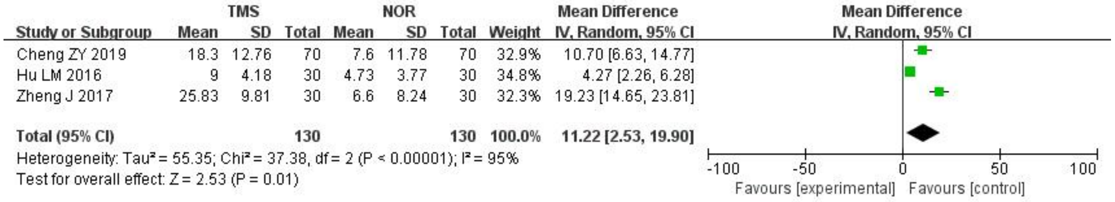

Study or Subgroup	tDCS Mean	tDCS SD	tDCS Total	NOR Mean	NOR SD	NOR Total	Weight	Mean Difference IV, Fixed, 95% CI
Jin J 2019	33.7	6.71	45	21.7	6.16	45	66.7%	12.00 [9.34, 14.66]
Mi Y 2019	24.66	7.62	15	19.67	8.86	15	13.5%	4.99 [-0.92, 10.90]
Song HY 2020	11.77	8.86	15	1.92	5.15	15	17.6%	9.85 [4.66, 15.04]
Yun GJ 2015	10.1	22.64	30	7.6	23.99	15	2.2%	2.50 [-12.10, 17.10]
**Total (95% CI)**			**105**			**90**	**100.0%**	**10.46 [8.29, 12.64]**

Heterogeneity: Chi² = 5.77, df = 3 (P = 0.12); I² = 48%
Test for overall effect: Z = 9.44 (P < 0.00001)

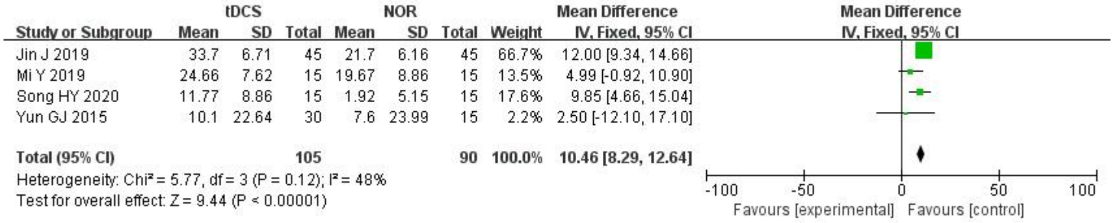

Study or Subgroup	VR Mean	VR SD	VR Total	NOR Mean	NOR SD	NOR Total	Weight	Mean Difference IV, Fixed, 95% CI
Faria AL 2016	6.3	29.87	9	2	26.83	9	0.2%	4.30 [-21.93, 30.53]
Faria AL 2018	5	24.06	12	0	44	12	0.2%	5.00 [-23.37, 33.37]
Guo JQ 2020	17.14	3.73	25	11	2.98	25	46.7%	6.14 [4.27, 8.01]
Ren YG 2020	21.99	5.97	40	17.68	5.67	40	25.1%	4.31 [1.76, 6.86]
Xiao X 2019	21.77	10.76	16	11.18	12.28	18	2.7%	10.59 [2.85, 18.33]
Zhang HL 2019	6.87	5.21	34	1.83	5.54	34	25.0%	5.04 [2.48, 7.60]
**Total (95% CI)**			**136**			**138**	**100.0%**	**5.52 [4.24, 6.80]**

Heterogeneity: Chi² = 3.08, df = 5 (P = 0.69); I² = 0%
Test for overall effect: Z = 8.46 (P < 0.00001)

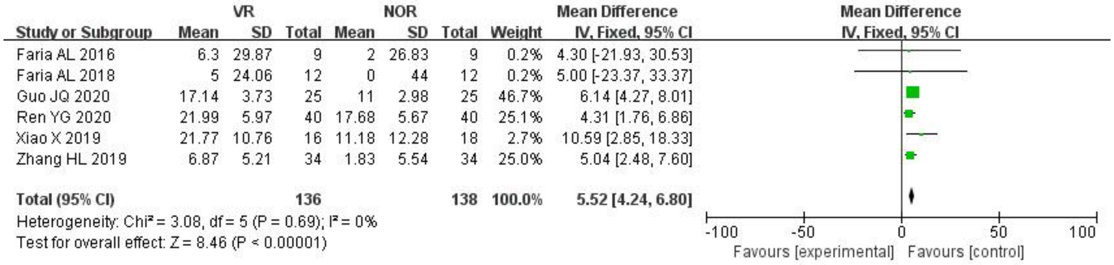

Study or Subgroup	CA Mean	CA SD	CA Total	NOR Mean	NOR SD	NOR Total	Weight	Mean Difference IV, Fixed, 95% CI
Chen JJ 2019	18.13	11.02	24	11.04	13.32	24	14.8%	7.09 [0.17, 14.01]
Liu RF 2019	17.74	11.68	15	4.13	11.56	15	10.3%	13.61 [5.29, 21.93]
Lu CH 2019	5.6	7.37	50	1.4	8.76	50	70.5%	4.20 [1.03, 7.37]
Xiao ST 2018	16.83	23.25	30	16.17	27.04	30	4.4%	0.66 [-12.10, 13.42]
**Total (95% CI)**			**119**			**119**	**100.0%**	**5.44 [2.78, 8.11]**

Heterogeneity: Chi² = 5.05, df = 3 (P = 0.17); I² = 41%
Test for overall effect: Z = 4.00 (P < 0.0001)

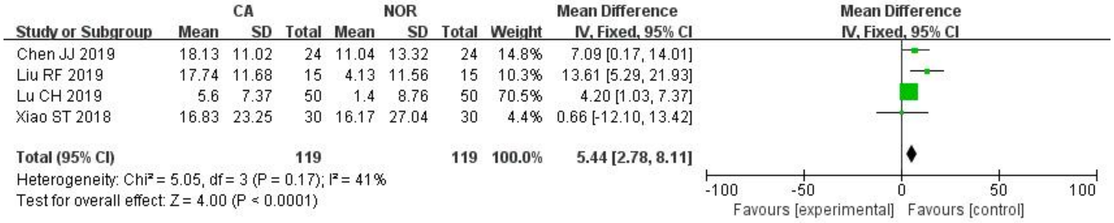

Study or Subgroup	Acupuncture Mean	Acupuncture SD	Acupuncture Total	NOR Mean	NOR SD	NOR Total	Weight	Mean Difference IV, Random, 95% CI
Chen AZ 2020	34.39	10.75	30	24.41	9.32	30	12.8%	9.98 [4.89, 15.07]
Ding X 2016	22.25	13.22	40	11.41	16.43	46	11.3%	10.84 [4.57, 17.11]
Mao CX 2019	38.32	5.15	39	23.9	5.67	39	15.9%	14.42 [12.02, 16.82]
Wang W 2018	28.23	8.96	50	17.58	7.74	50	15.0%	10.65 [7.37, 13.93]
Yang M 2017	19.28	9.92	47	12.22	9.84	44	14.1%	7.06 [3.00, 11.12]
Yang Y 2019	33.94	7.71	40	20.84	7.08	40	15.1%	13.10 [9.86, 16.34]
Zhang HL 2019	4.93	5.37	34	1.83	5.54	34	15.7%	3.10 [0.51, 5.69]
**Total (95% CI)**			**280**			**283**	**100.0%**	**9.86 [6.22, 13.50]**

Heterogeneity: Tau² = 20.16; Chi² = 45.89, df = 6 (P < 0.00001); I² = 87%
Test for overall effect: Z = 5.31 (P < 0.00001)

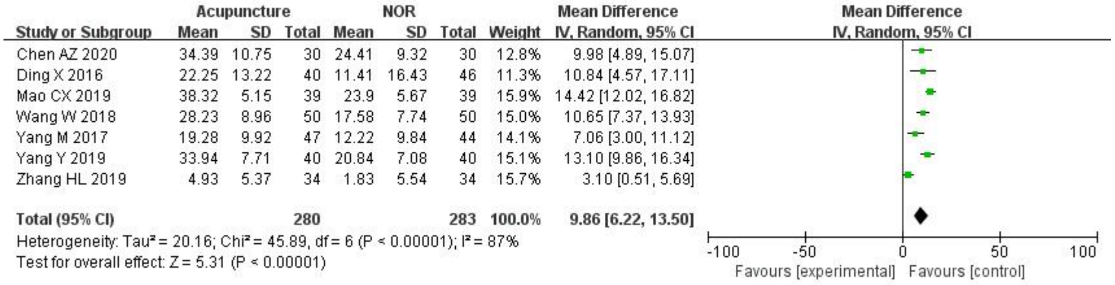

Study or Subgroup	VR Mean	VR SD	VR Total	Acupuncture Mean	Acupuncture SD	Acupuncture Total	Weight	Mean Difference IV, Random, 95% CI
Zhang HL 2019	6.87	5.21	34	4.93	5.37	34	100.0%	1.94 [-0.57, 4.45]
**Total (95% CI)**			**34**			**34**	**100.0%**	**1.94 [-0.57, 4.45]**

Heterogeneity: Not applicable
Test for overall effect: Z = 1.51 (P = 0.13)

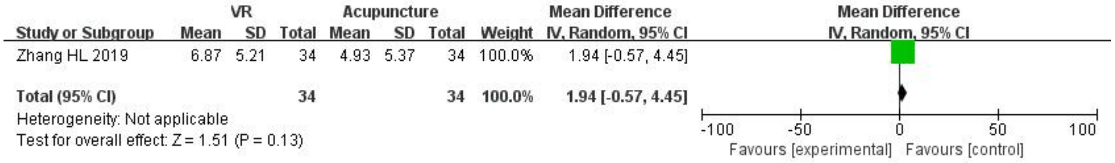

Supplementary Figure 3. Forest plots of the pairwise meta-analysis for BI. TMS: Transcranial Magnetic Stimulation; VR: Virtual Reality Exposure Therapy; CA: Computer-assisted cognitive rehabilitation; tDCS: Transcranial Direct Current Stimulation; Acu: Acupuncture; NOR: Normal rehabilitation, including conventional rehabilitation and routine cognition training.

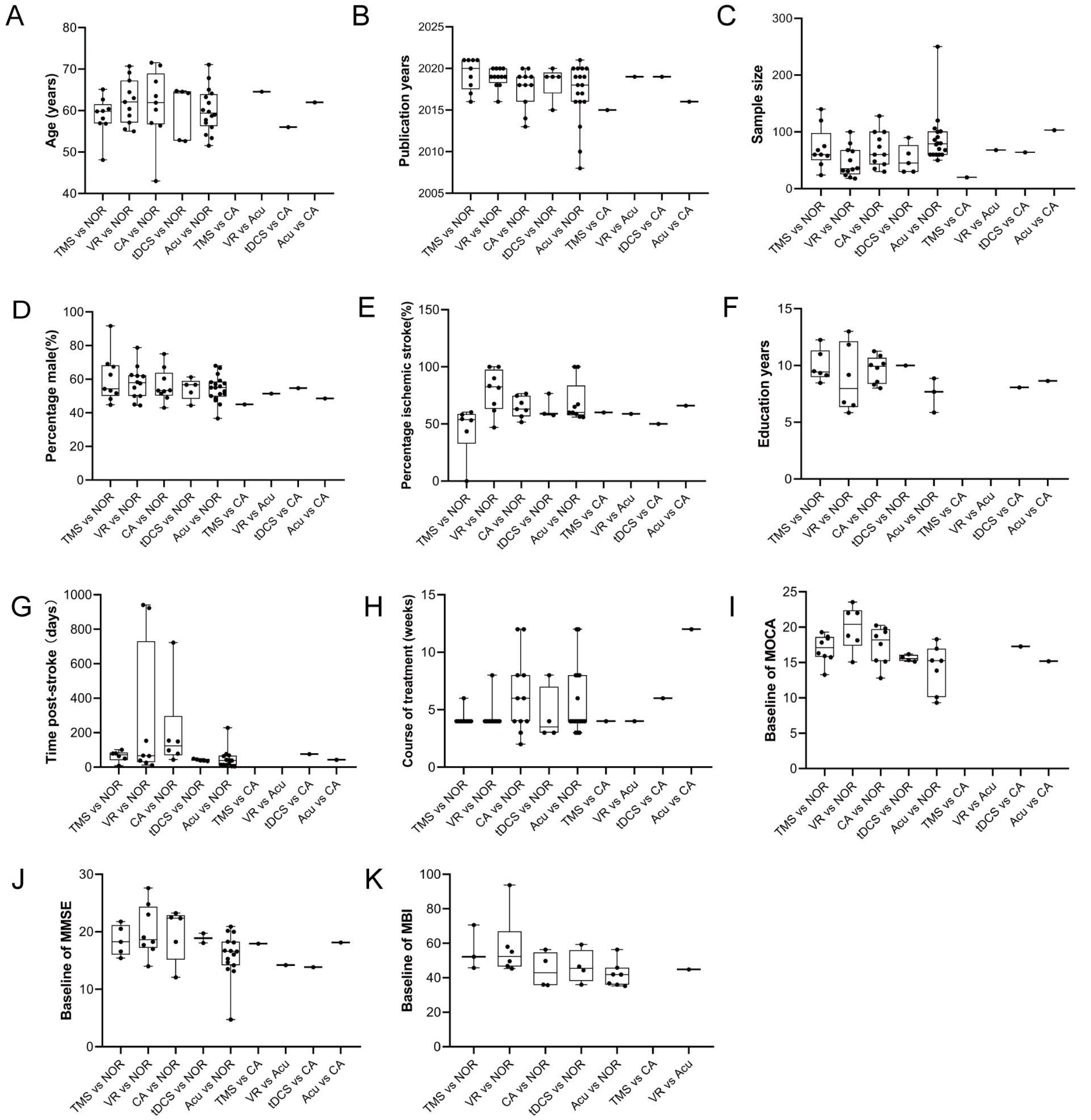

Supplementary Figure 4. Assessment of transitivity. (A) Age, (B) Pulication year, (C) Sample size, (D) Percentage of male, (E) Percentage of ischemic stroke, (F) Education year, (G) Time post-stroke, (H) Course of treatment, (I) Baseline of MoCA, (J) Baseline of MMSE, (K) Baseline of BI. TMS: Transcranial Magnetic Stimulation; VR: Virtual Reality Exposure Therapy; CA: Computer-assisted cognitive rehabilitation; tDCS: Transcranial Direct Current Stimulation; Acu: Acupuncture; NOR: Normal rehabilitation, including conventional rehabilitation and routine cognition training.

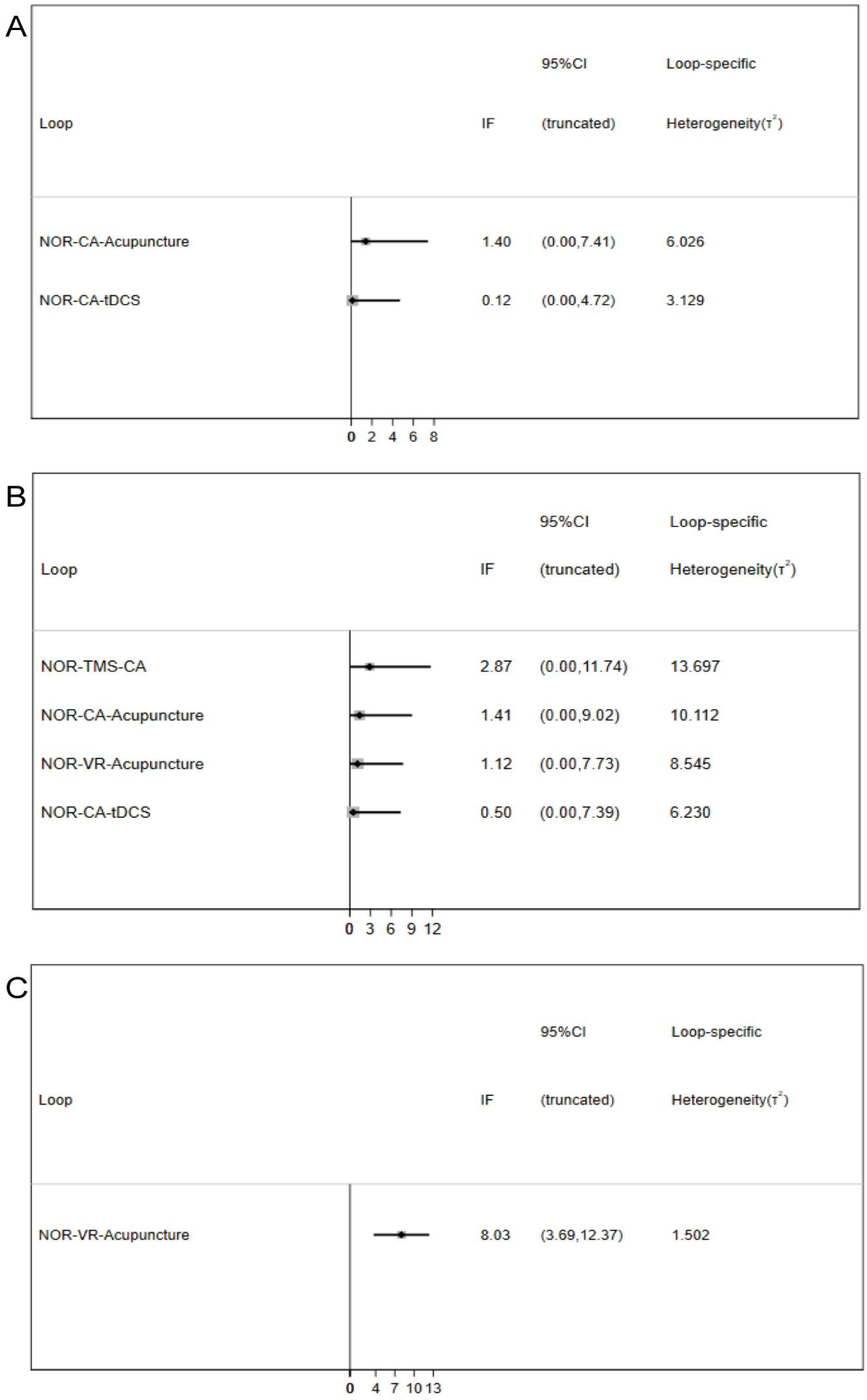

Supplementary Figure 5. Assessment of inconsistency (Loop-specific estimation). (A) Moca, (B) MMSE, (C) BI. TMS: Transcranial Magnetic Stimulation; VR: Virtual Reality Exposure Therapy; CA: Computer-assisted cognitive rehabilitation; tDCS: Transcranial Direct Current Stimulation; Acu: Acupuncture; NOR: Normal rehabilitation, including conventional rehabilitation and routine cognition training.

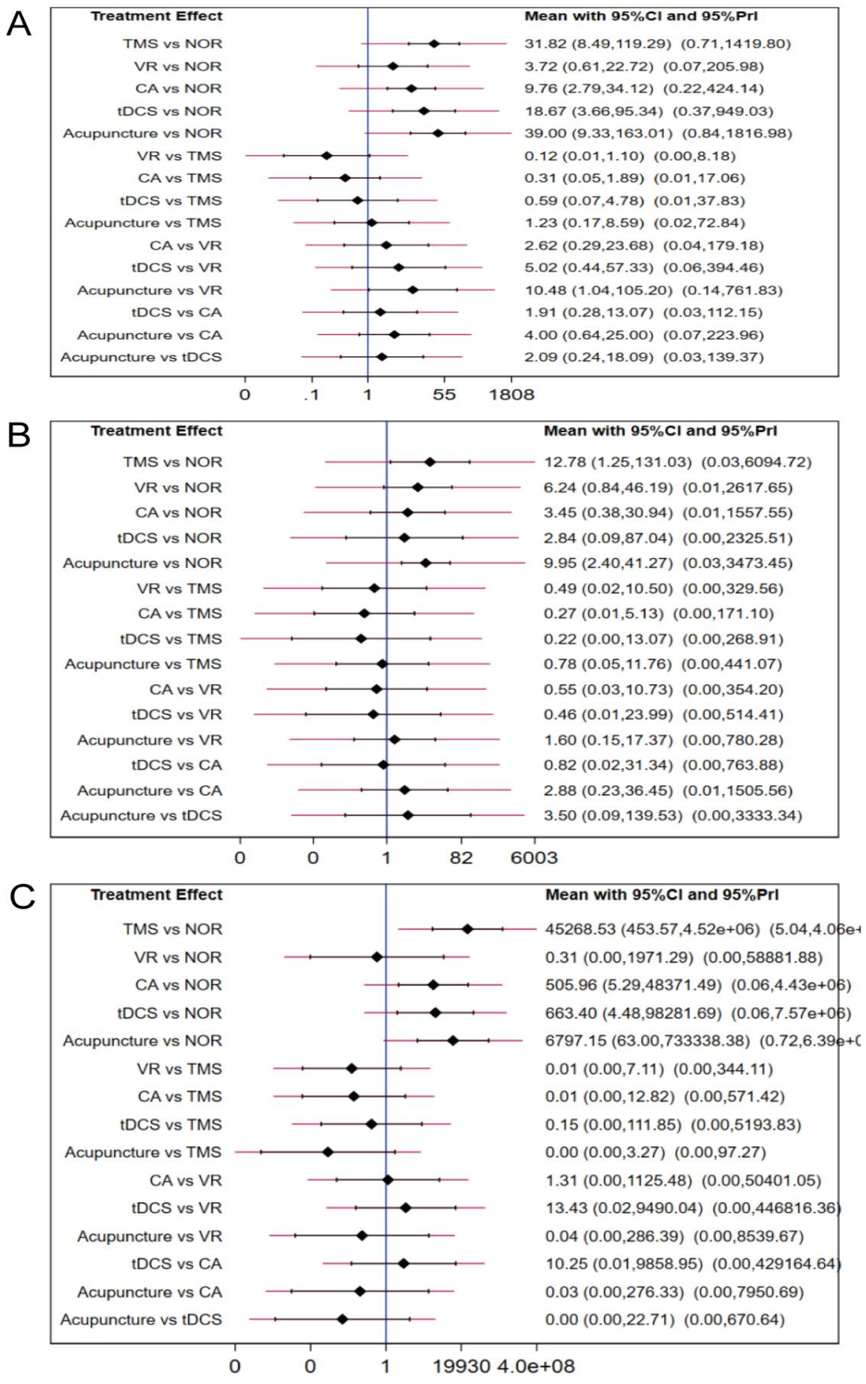

Supplementary Figure 6. The predictive interval between fixed and random (Assessment of heterogeneity). (A) Moca, (B) MMSE, (C) BI. TMS: Transcranial Magnetic Stimulation; VR: Virtual Reality Exposure Therapy; CA: Computer-assisted cognitive rehabilitation; tDCS: Transcranial Direct Current Stimulation; Acu: Acupuncture; NOR: Normal rehabilitation, including conventional rehabilitation and routine cognition training.

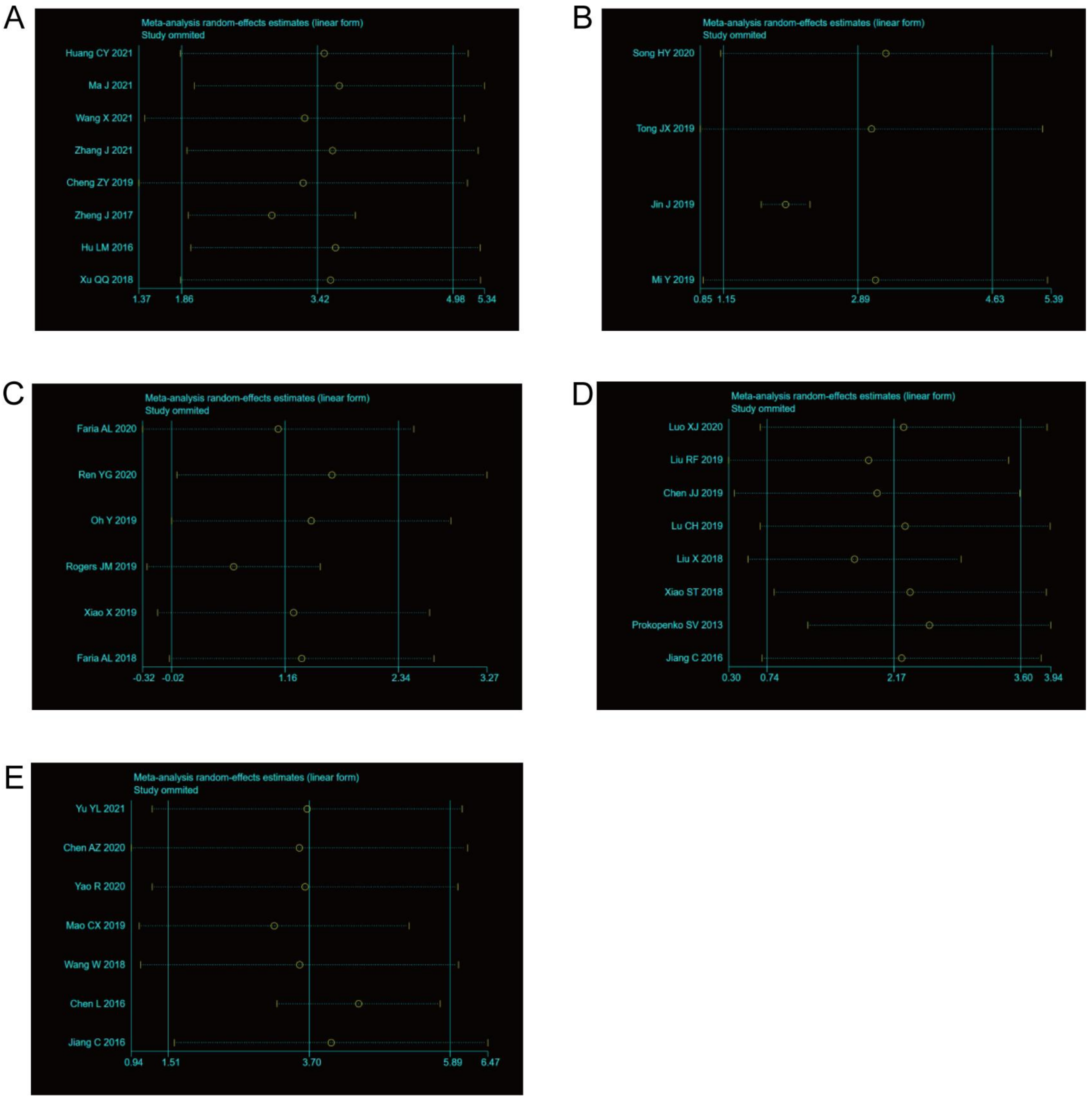

Supplementary Figure 7. Sensitivity analyses of MOCA. (A) TMS vs NOR, (B) tDCS vs NOR, (C) VR vs NOR, (D) CA vs NOR, (E) Acu vs NOR. TMS: Transcranial Magnetic Stimulation; VR: Virtual Reality Exposure Therapy; CA: Computer-assisted cognitive rehabilitation; tDCS: Transcranial Direct Current Stimulation; Acu: Acupuncture; NOR: Normal rehabilitation, including conventional rehabilitation and routine cognition training.

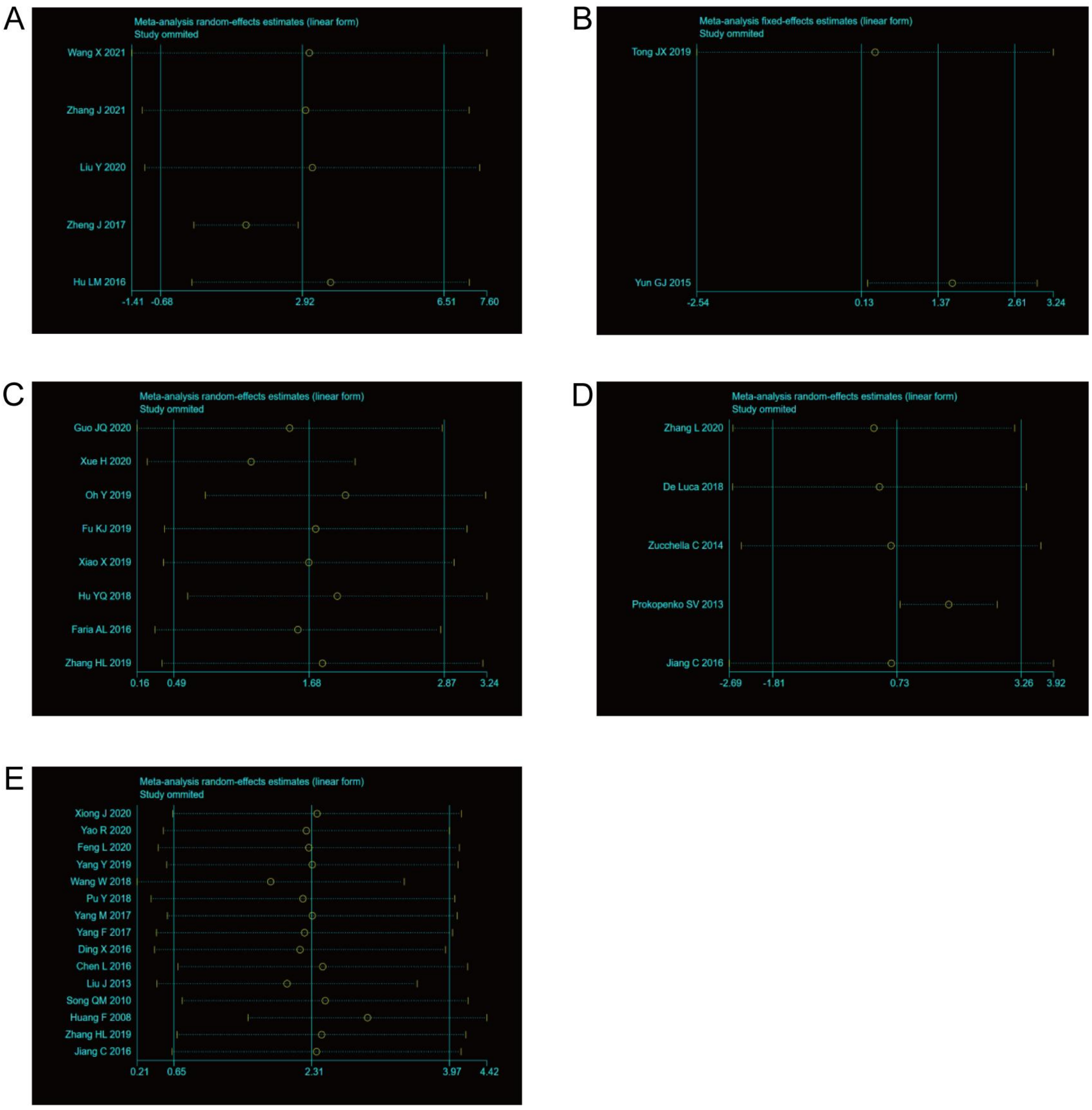

Supplementary Figure 8. Sensitivity analyses of MMSE. (A) TMS vs NOR, (B) tDCS vs NOR, (C) VR vs NOR, (D) CA vs NOR, (E) Acu vs NOR. TMS: Transcranial Magnetic Stimulation; VR: Virtual Reality Exposure Therapy; CA: Computer-assisted cognitive rehabilitation; tDCS: Transcranial Direct Current Stimulation; Acu: Acupuncture; NOR: Normal rehabilitation, including conventional rehabilitation and routine cognition training.

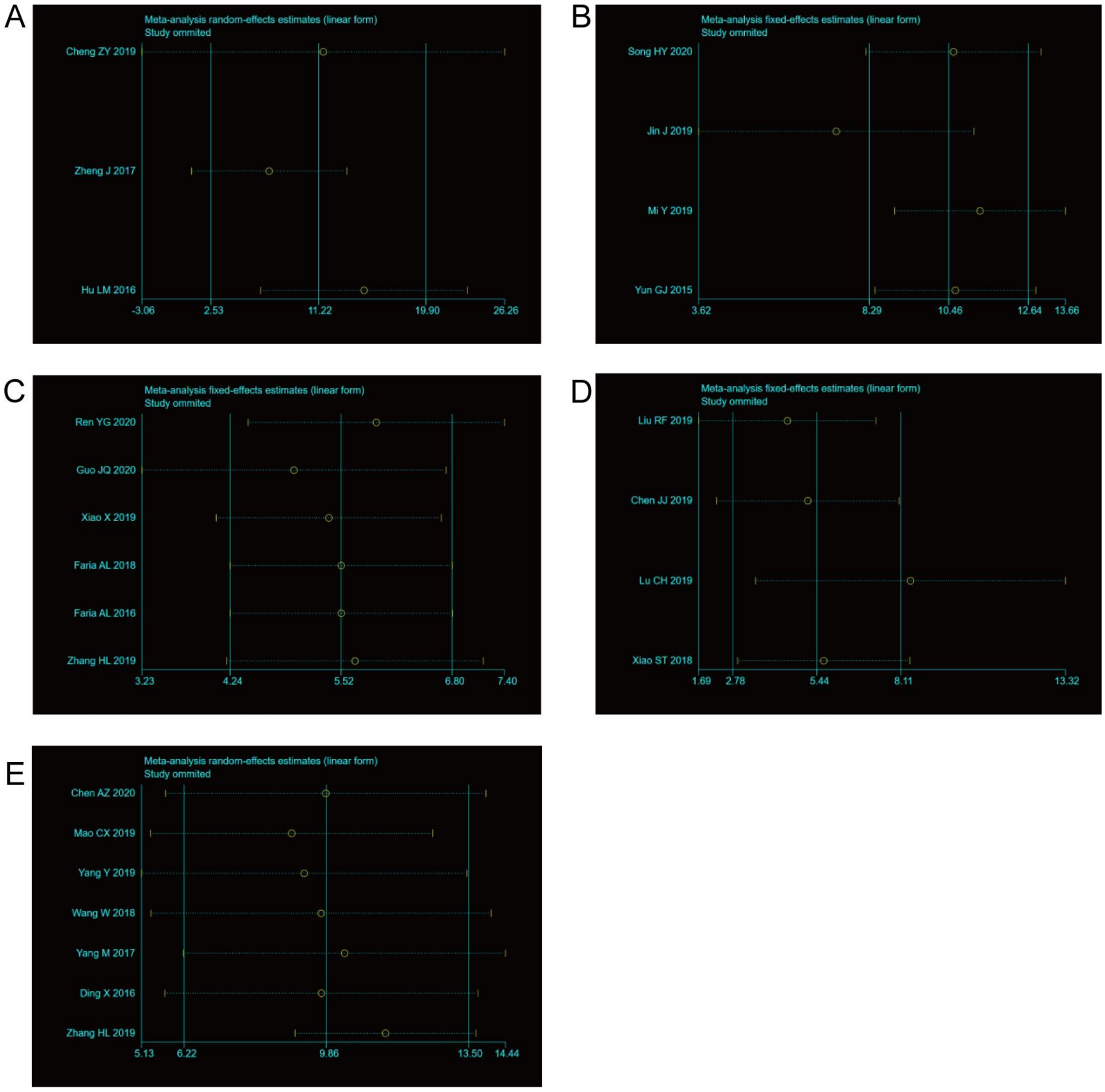

Supplementary Figure 9. Sensitivity analyses of BI. (A)TMS vs NOR, (B) tDCS vs NOR, (C)VR vs NOR, (D) CA vs NOR, (E)Acu vs NOR. TMS: Transcranial Magnetic Stimulation; VR: Virtual Reality Exposure Therapy; CA: Computer-assisted cognitive rehabilitation; tDCS: Transcranial Direct Current Stimulation; Acu: Acupuncture; NOR: Normal rehabilitation, including conventional rehabilitation and routine cognition training.
